# Comparative transcriptomic analysis unveils interactions between the regulatory CarS protein and light response in *Fusarium*

**DOI:** 10.1186/s12864-019-5430-x

**Published:** 2019-01-21

**Authors:** Macarena Ruger-Herreros, Obdulia Parra-Rivero, Javier Pardo-Medina, Francisco J. Romero-Campero, M. Carmen Limón, Javier Avalos

**Affiliations:** 1 Department of Genetics, Faculty of Biology, University of Seville, E-41012 Seville, Spain; 2 Department of Computer Science and artificial Intelligence, University of Seville, E-41012 Seville, Spain; 3 Plant Development Unit, Institute for Plant Biochemistry and Photosynthesis, University of Seville – CSIC, E-41012 Seville, Spain

**Keywords:** RNA-Seq, Light, CarS, RING-finger protein, Transcriptomics, Oxidative stress, Photoinduction

## Abstract

**Background:**

The orange pigmentation of the agar cultures of many *Fusarium* species is due to the production of carotenoids, terpenoid pigments whose synthesis is stimulated by light. The genes of the carotenoid pathway and their regulation have been investigated in detail in *Fusarium fujikuroi*. In this and other *Fusarium* species, such as *F. oxysporum*, deep-pigmented mutants affected in the gene *carS,* which encodes a protein of the RING-finger family, overproduce carotenoids irrespective of light. The induction of carotenogenesis by light and its deregulation in *carS* mutants are achieved on the transcription of the structural genes of the pathway. We have carried out global RNA-seq transcriptomics analyses to investigate the relationship between the regulatory role of CarS and the control by light in these fungi.

**Results:**

The absence of a functional *carS* gene or the illumination exert wide effects on the transcriptome of *F. fujikuroi*, with predominance of genes activated over repressed and a greater functional diversity in the case of genes induced by light. The number of the latter decreases drastically in a *carS* mutant (1.1% vs. 4.8% in the wild-type), indicating that the deregulation produced by the *carS* mutation affects the light response of many genes. Moreover, approximately 27% of the genes activated at least 2-fold by light or by the *carS* mutation are coincident, raising to 40% for an 8-fold activation threshold. As expected, the genes with the highest changes under both regulatory conditions include those involved in carotenoid metabolism. In addition, light and CarS strongly influence the expression of some genes associated with stress responses, including three genes with catalase domains, consistent with roles in the control of oxidative stress. The effects of the CarS mutation or light in the transcriptome of *F. oxysporum* were partially coincident with those of *F. fujikuroi*, indicating the conservation of the objectives of their regulatory mechanisms.

**Conclusions:**

The CarS RING finger protein down-regulates many genes whose expression is up-regulated by light in wild strains of the two investigated *Fusarium* species, indicating a regulatory interplay between the mechanism of action of the CarS protein and the control by light.

**Electronic supplementary material:**

The online version of this article (10.1186/s12864-019-5430-x) contains supplementary material, which is available to authorized users.

## Background

The genus *Fusarium* comprises a large group of saprophytic and phytopathogenic filamentous fungi, widely distributed in all terrestrial ecosystems. Some of them are well-known research models for basic biological processes, such as *Fusarium oxysporum* in studies of plant pathogenesis [1], or *Fusarium fujikuroi* because of its ability to produce gibberellins, growth-promoting plant hormones [2]. Like many other species of *Fusarium*, *F. fujikuroi* is capable of producing a large array of secondary metabolites in addition to gibberellins, including bikaverin [3], fusarins [4], fusaric acid [4], fusarubin [5], and carotenoids, among others [6]. Recently, the analysis of its genome and the deduced proteome uncovered an unexpected complexity for its secondary metabolism [7]. In recent years *F. fujikuroi* has become a preferred fungal model to investigate the biochemical basis for the biosynthesis and regulation of several of these compounds, such as gibberellins, bikaverin [8], and carotenoids [9].

The synthesis of secondary metabolites is usually modulated by external cues, such as light or nutrient availability. Light is an environmental signal used by fungi to control many aspects of their biology and life cycle [10]. Light also serves as an indicator of potentially hazardous stresses, such as exposure to air, desiccation and osmotic stress, heat shock, or UV exposure. In *Fusarium sp.*, light stimulates the synthesis of carotenoids [11]. Thus, when dark-grown cultures are illuminated, carotenoids accumulate for several hours after light onset in *Fusarium aquaeductuum* [12] and *F. fujikuroi* [13], providing a characteristic orange pigmentation. All the structural genes of the carotenoid pathway have been identified and their functions were determined by targeted mutation or by biochemical assays [14]. The *carRA*, *carB*, *carT*, and *carD* genes encode a bifunctional cyclase/phytoene synthase, a desaturase, a torulene-cleaving dioxygenase, and an aldehyde dehydrogenase, respectively, involved in the sequential biosynthetic steps needed to produce the acid xanthophyll neurosporaxanthin (Additional file 1: Figure S1). The *carRA* and *carB* genes are linked in a cluster with the retinal-forming dioxygenase *carX* gene and with the *carO* rhodopsin gene [15]. Except *carD*, only moderately induced by light, *carT* and the *carRA*/c*a*rB/*carX*/*carO* cluster are strongly photoregulated [9].

Expression studies revealed that stimulation of carotenogenesis by light is achieved at the level of transcription, with a rapid increase in mRNAs of most of the structural genes during the first hour of illumination [11]. The dependence on wavelength of carotenoid photoinduction was investigated in *F. aquaeductuum*, showing an action spectrum consistent with the participation of a flavoprotein [16]. In *Neurospora crassa*, a fungus with a similar light-regulated carotenoid pathway, the photoreceptor that mediates this response is the White Collar complex (WCC), a heterodimer formed by the flavin photoreceptor WC-1 and its partner WC-2 [17]. Upon illumination, the WCC is activated by light and binds to the promoters of target photo-regulated genes to stimulate their transcription. The WCC is also responsible for other processes stimulated by light in *N. crassa*, and photocarotenogenesis is totally abolished in the absence of either of the two White Collar (WC) proteins [18].

The *wc-1* ortholog gene has been investigated in *F. fujikuroi* [19] and in *F. oxysporum* [20], where it was named *wcoA* and *wc1*, respectively. In both species, the null mutants for this gene retain a certain level of photoinduction of carotenoids when grown under continuous light, indicating that the WC flavoprotein is not the only photoreceptor involved in photocarotenogenesis. A more detailed analysis has shown that the stimulation of carotenoid biosynthesis by light in *F. fujikuroi* is carried out in two stages, a fast one mediated by WcoA and a slower one mediated by another flavin photoreceptor, the DASH cryptochrome CryD [21]. The significant carotenoid accumulation in *wcoA* mutants under constant illumination can be attributed to CryD, whose photoactivity has been demonstrated experimentally [22]. Interestingly, the mutation of *wcoA* or *cryD* also affects the production of other secondary metabolites [19, 23], even in the dark in the case of *wcoA*, indicating other functions for these photoreceptors, in addition to the control of carotenogenesis.

Deep-orange mutants, which produce large amounts of neurosporaxanthin and other carotenoids, have been described in *F. fujikuroi* [24] and *F. oxysporum* [25]. Their increased carotenoid content is due to the enhanced expression of the structural genes *carRA*, *carB* [15, 26], *carT* [27], and *carD* [28] either in the light or in darkness. However, mRNA levels for the *car* genes still increase in response to light in the *carS* mutants of *F. fujikuroi* [27] or *F. oxysporum* [25]. As observed for *wcoA*, the *carS* mutation not only affects the synthesis of carotenoids, but also the production of other secondary metabolites, such as gibberellins or bikaverin [29, 30].

The gene *carS* encodes a protein of the RING finger (RF) family that includes a putative LON protease domain [25, 31]. This structural combination was previously described in the CrgA protein of the Zygomycota fungus *M. circinelloides*, whose mutants exhibit a similar pattern of overproduction of carotenoids [32]. RING-finger domains are characteristic of a family of ubiquitin ligases, which transfer ubiquitin to specific target proteins to modulate their activity or determine their fate. In the case of CrgA, its ability to inactivate MCWC-1b white collar protein through its specific mono- and di-ubiquitylation was demonstrated [32]. The molecular mechanism by which the CarS protein down-regulates the genes for carotenoid biosynthesis in *Fusarium* has not yet been investigated. However, although the *Fusarium* and *Mucor* species are taxonomically very distant, and the CarS and CrgA polypeptides exhibit considerable sequence divergence, the conservation of the structural domains suggests similar mechanisms of action.

To better understand the regulatory role of the CarS protein in *F. fujikuroi* and its possible relationship with the regulation by light, we have carried out ribonucleic acid sequencing (RNA-seq) analyses from mycelia of the wild type, a *carS* mutant and a *carS-*complemented strain, grown in the dark or exposed to light for 1 hour. The analyses were extended to the effects of light and mutation of *carS* in *F. oxysporum*. The results revealed that CarS and light control the expression of a large battery of genes in both species, among which those of the *car* cluster stand out for the magnitude of their response. Interestingly, the data reveal an outstanding overlap between the genes induced by light and derepressed in the *carS* mutant, suggesting regulatory relationships between CarS and light. Some of the commonly regulated genes are supposedly involved in oxidative stress.

## Results

### Experimental design and analysis of global transcriptomic data

**Fig. 1:**
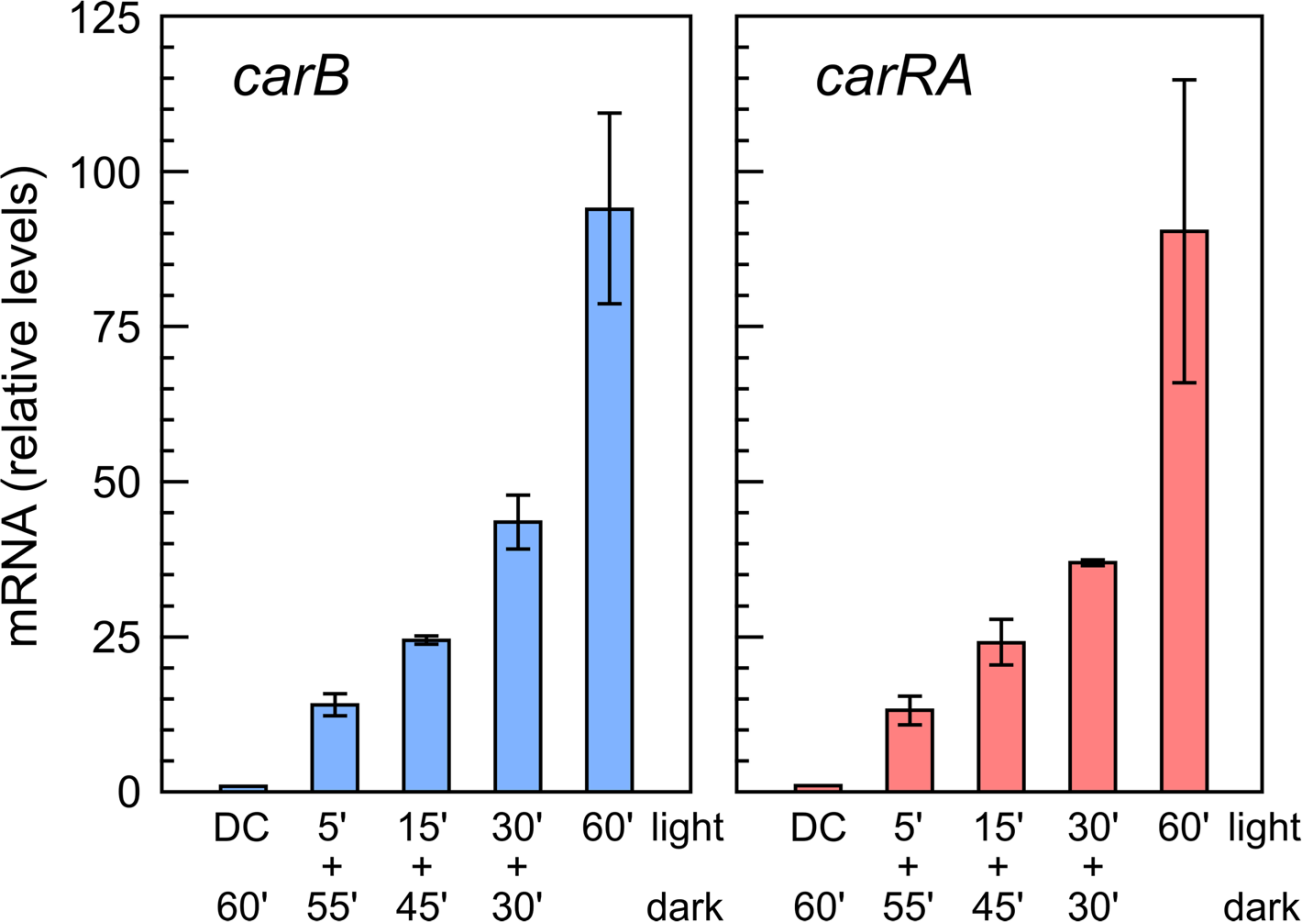
Effect of duration of light pulse on transcript levels of *carRA* and *carB* genes. RT-PCRs of genes *carRA* and *carB* were carried out in total RNA samples from the wild type grown in the dark and illuminated for 5 min, 15 min, 30 min, or 1 h. To make the results comparable, in the dark control (DC) and in the samples exposed to light pulses of less than 1 hour, the incubation was extended up to 1 hour in the dark, as indicated in the column labels. Data are referred to the data in the dark and show the mean and error of the mean of three biological replicates

Previous analyses showed low mRNA levels of the genes of the carotenoid pathway in the dark-grown mycelia and a rapid accumulation after illumination, reaching maximum levels after approximately 1 hour of exposure to light [11]. To better understand the experimental conditions, we verified whether shorter exposures to light allow a maximum response after 1 hour of incubation. Therefore, real-time q-PCR experiments, abbreviated RT-qPCR hereafter, were done comparing wild type mycelia treated with different light pulses with a total incubation of 60 min (5 min light + 55 min dark; 15 min light + 45 min dark, 30 min light + 30 min dark and 60 min light). The results showed that mRNA levels of the *carRA* and *carB* genes, used as controls for photoinduction, increased gradually with longer light exposures, reaching their higher levels after 1 hour of illumination (Fig. 1). Therefore, to check the effect of light on the transcriptome of *F. fujikuroi*, we compared dark cultures with parallel cultures exposed for 1 hour to light. To examine the effect of the *carS* mutation, we used the mutant SG39, which was previously found to have a mutation that changes a highly conserved cysteine residue of the CarS protein [31]. As an additional control, we used SG256, a stable *carS*^*+*^ complemented strain obtained in two steps from a SG39 transformant [31] (Additional file 1: Figure S2).

The basic parameters of the RNA-seq data generated in this study, such as G + C content, millions of raw reads and percentage of mapped reads, are presented in Additional file 2: Table S1. In the genome of *F. fujikuroi* [7], the annotated coding sequences account for 49.2% of the total sequence, with an average coding length of 1457 nucleotides, an average number of 2.8 exons per gene and an average for the length of the exons of 518 nucleotides. The overall G + C content is 47.4%, which rises to 51.5% in the protein coding sequences. The sequences obtained in our RNA-seq study had an average G + C content close to 52%, in good agreement with the overall value of the genome for the coding sequences. With respect to read quality, its analysis with the FastQC package yielded similar high-quality profiles for all the samples (representative example in Additional file 1: Figure S3).

Mapping of the raw sequence data to the annotated reference genome downloaded from Ensembl Fungi (http://fungi.ensembl.org/index.html) identified transcripts for 15,095 genes, with 15,106 isoforms, and assignable to 14,815 coding sequences. These numbers fit well with the number of identified transcription start sites, 15,101. Once a high read quality was verified, the gene expression levels in the different samples were calculated for each gene using the *Tuxedo* protocol (see Materials and Methods). The read counts for each gene were obtained from the mapping results and normalized to FPKM values (fragments per kilobase of exon per million of mapped reads), and the overall distribution of gene expression in each sample was represented in boxplots (Additional file 1: Figure S4), which showed comparable gene expression patterns between the different samples. In addition, the representation of the coefficient of variation between replicates as a function of the expression levels (FPKM) showed a similar distribution in all strains and conditions, with a higher variation for genes with low expression, and an increasing reproducibility for genes with higher expression levels (Additional file 1: Figure S5). Therefore, the mean of the two biological replicates was used hereafter as the expression value in each strain and experimental condition.

### Effects of light and *carS* mutation on the *F. fujikuroi* transcriptome

**Fig. 2:**
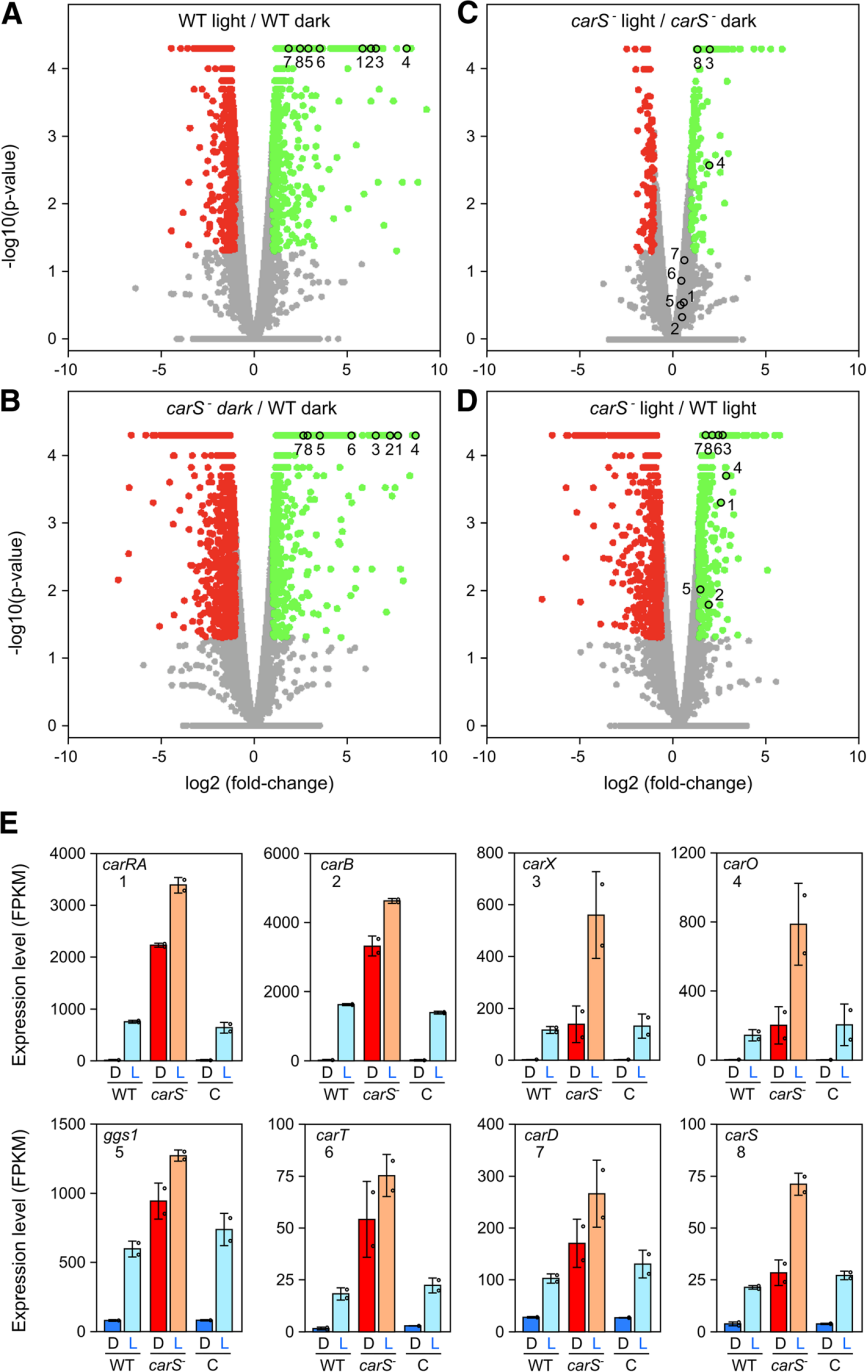
Effect of light and *carS* mutation on transcript levels of the *F. fujikuroi* genes. **a-d** Volcano plot representations of global expression data in the indicated pairwise comparisons. The empty circles indicate the positions of the *car* genes shown in panel E. **a** Effect of light in the wild type (wild type in the dark vs. wild type after illumination). **b** Effect of the SG39 genotype in the dark (wild type in the dark vs. *carS* mutant SG39 in the dark). **c** Effect of light in the *carS* mutant (*carS* mutant SG39 in the dark vs. *carS* mutant SG39 after illumination). **d** Effect of the SG39 genotype after illumination (wild type after illumination vs. *carS* mutant SG39 after illumination). **e** Expression levels of the *car* genes in the wild type (WT), *carS* mutant SG39 (*carS*^*−*^) and complemented strain SG256 (C) in the dark (D) or after one-hour illumination (L)

The differentially expressed genes were determined using a two-fold change threshold and a *p*-value threshold of 0.05 when comparing each condition with its control. The numbers and proportions of the genes activated or repressed in response to light or in the *carS* mutant SG39, in the different combinations of interest, are summarized in Table 1. The light and the SG39 genotype had similar impacts on the transcriptome, with at least 4–5% of genes with 2× alterations up or down in their expression. The effect of light and SG39 genotype over the entire transcriptome was represented by volcano plots (Fig. 2) and scatter plots (Additional file 1: Figure S6). In the wild type, the number of genes activated by light was slightly higher than those repressed by light (Table 1), but the graphical representations revealed a predominance of activation among the genes with the largest changes in their expression (green dots in Fig. 2a and Additional file 1: Figure S6A). This is more clearly seen in a graphical representation of the 30 genes with the largest activating or repressing fold-changes (blue arrowheads in Additional file 1: Figure S7A). This indicates a more relevant up-regulating role for light in *Fusarium*. A similar result was noticeable in the case of the effect of the SG39 genotype in the dark: although the number of genes with at least a 2-fold change in their mRNA levels was even greater for those with a decreased expression in this mutant strain (821 vs 716, Table 1), genes with major changes were more abundant among those up-regulated (green dots in Fig. 2b and Additional file 1: Figure S6B; Additional file 1: Figure S7A).

**Table 1:** Number of genes whose expression changes more than two-fold above (activated) or below (repressed) the controls, under the indicated conditions

	Activated	%	Repressed	%
Effect of light in the wild type	724	4.80	535	3.54
Effect of light in the *carS* mutant SG39	206	1.36	132	0.87
Effect of light in the complemented strain SG256	876	5,80	244	1,62
Effect of the SG39 genotype in the dark^a^	717	4.75	821	5.44
Effect of the SG39 genotype in the light^a^	509	3.37	633	4.19

The percentage of the total number of genes detected in the analysis is indicated to the right of each value

^a^Comparisons between SG39 mutant and wild strain of *F. fujikuroi*

The number of genes influenced by light was much lower in the *carS* mutant SG39 (285 genes, Table 1) than in the wild strain (1259 genes) or the complemented strain SG256 (1120 genes). In addition, large changes in mRNA levels resulting from illumination were very scarce in the *carS* mutant (Fig. 2c and Additional file 1: Figure S6C). This suggests that the deregulation produced by the *carS* mutation in many genes in SG39 affects their ability to respond to light. This is also visualized in Volcano and scatter plots that show the effect of the *carS* mutation in the light (Fig. 2d and Additional file 1: Figure S6D). The number of genes activated or repressed in the *carS* mutant SG39 is sensibly reduced in light compared to darkness (509 vs 717 for up-regulation and 633 vs 821 for down-regulation, Table 1). In this case there is a marked reduction in genes with high induction in expression levels (compared green dots in Fig. 2b and d, and in Additional file 1: Figure S6B and D).

The expression data are very reproducible for genes more drastically influenced by light or by SG39 genotype. This is the case of the structural genes of carotenoid metabolism *ggs1*, *carRA*, *carB*, *carT*, *carD*, and *carX,* as well as the *carO* rhodopsin gene or the *carS* regulatory gene (empty circles in Fig. 2a and b). However, the relative impact of light or the *carS* mutation on these genes is mostly attenuated when the two regulatory conditions act at the same time (Fig. 2c and d). The *car* genes differ in their absolute expression levels but exhibit a similar regulatory pattern in response to light and *carS*^*−*^ genotype (Fig. 2e).

Since it is likely that the *carS* mutant SG39 has other unrelated mutations, only those genes that recovered a wild-type expression level in the complemented strain SG256 were hereafter considered as affected by the *carS* mutation. To visualize the effect of *carS* complementation in the SG39 genetic background, we compared in Venn diagrams the sets of genes activated or repressed in SG39 with those with the opposite effect in SG256 (Additional file 1: Figure S8). This comparison showed that approximately 55% of the genes whose expression changed in SG39 recovered their basal expression in SG256, suggesting that many genes with changes in expression close to the 2× threshold were not related to the *carS* mutation but to random variations. A comparison on the effect of light on the wild type and SG256 showed a greater overlap for the genes induced by light than for those repressed by light (64% vs. 21%, referred to the number of genes regulated by light in the wild-type), suggesting a greater abundance of changes in gene expression due to random variations in the set of genes repressed by light.

### Relationship between the effects of light and *carS* mutation

**Fig. 3:**
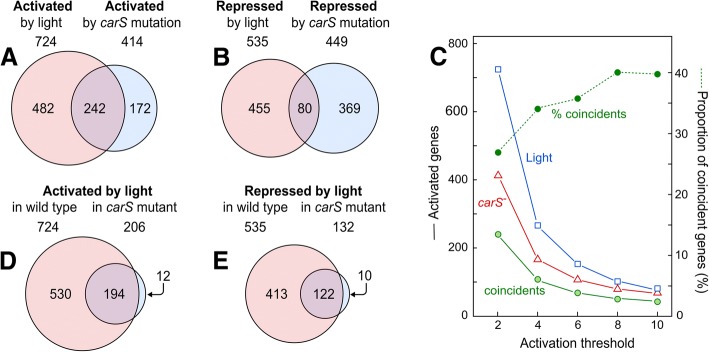
Overlap between the effect of light and *carS* mutation in the transcriptome of *F. fujikuroi.* **a**-**b** Venn diagrams of *F. fujikuroi* genes activated or repressed by light or by the *carS* mutation. The effect of the *carS* mutation was corrected with the data of the SG256 complemented strain. **c**-**d** Venn diagrams of *F. fujikuroi* genes activated or repressed by light in the wild type or in the *carS* mutant SG39. In all the diagrams, the numbers indicate genes that correspond to the conditions mentioned above. The surfaces of the circles are proportional to the numbers of genes. Overlapping areas of the circles correspond to genes that coincide in the conditions compared. The sets of genes activated/repressed by the *carS* mutation in dark conditions overlap significantly with the sets of genes activated/repressed in the wild type (WT) by light according to *p*-values < 2.2 × 10^− 16^ and odds ratios of 12.18 and 5.03, respectively, calculated using Fisher’s exact test. **e** Effect of the threshold for expression change in the number of genes activated by light and by the *carS* mutation. The graph represents the genes activated by any of the two conditions and the coinciding ones. Percentages of coinciding genes are also represented (dotted line)

The sets of genes that increase their expression as a consequence of light or the *carS* mutation showed a significant overlap (Fig. 3a). According to an enrichment analysis based on Fisher’s exact test, given that genome of *F. fujikuroi* consists of 15,095 genes, it would be expected that approximately 20 genes coincided by chance if both regulatory phenomena, light and *carS* mutation, were independent. Instead we observed 241 overlapping genes that resulted in a significant enrichment of approximately 12× with a *p*-value < 2.2 × 10^− 16^. There were also more genes coincident between those repressed by light or the *carS* mutation (80) than expected by chance (15, Fig. 3b). Therefore, the data show a patent relationship between the genes activated or repressed by light and those activated or repressed in the *carS* mutant. However, overlaps were mostly non-existent in comparisons between sets of genes activated by light and repressed by the *carS* mutation, or vice versa (Additional file 1: Figure S9A and B), with matching gene numbers as expected by chance. When higher activation thresholds - up to 10× - were considered, the degree of coincidence between the sets of genes activated by light and the *carS* mutation increased to approximately 40% of the genes in both conditions (Fig. 3c).

As already mentioned, the number of genes activated by light was much lower in the *carS* mutant than in the wild strain. However, most of the genes that were still activated or repressed by light in the *carS* mutant were also activated or repressed by light in the wild type (Fig. 3d and e). These results indicate that there is a set of genes that are regulated by light independently of CarS function. On the other hand, no coincidence was detected when the sets of genes activated or repressed by light in one strain were compared with those that exhibited the opposite pattern in the other (Additional file 1: Figure S9C and D), which supports the alleged relationship between genes activated by light and by the *carS* mutation.

### Identity of strongly differentially expressed genes

The genes influenced by light or by the *carS* mutation were sorted by their degree of activation or repression and identified in the proteome of *F. fujikuroi* (complete list of genes exceeding 2-fold change displayed in Additional file 3: Table S2A-D). There was a considerable difference between the highest levels of activation and repression by light: while the most up-regulated genes in the wild strain increased their expression about 600 fold, the expression of the most repressed genes only decreased about 20-fold (Additional file 1: Figure S7A, Additional file 3: Table S2A and B). The differences between the maximum activations and repressions were less remarkable in the case of the effect of the *carS* mutation (Additional file 1: Figure S7A, Additional file 3: Table S2C and D). In the lists of genes activated by light or by the *carS* mutation, those of the *car* cluster (*carX*, *carRA*, *carB*, and *carO*, Additional file 1: Figure S1) were among the most activated, with *carO* standing out as the fourth gene in induction by light and the third gene in induction in the *carS* mutant (Additional file 1: Figure S7A, and Additional file 3: Table S2A and C). This result confirms the *car* cluster as one of the major regulatory targets of light and CarS. The effects of light and *carS* mutation on *car* genes are summarized in Table 2. In accordance with previous data [27, 28], the transcriptional changes found for the *carT* and *carD* structural genes, unlinked to the *car* cluster, were less pronounced. However, one of the most affected genes was FFUJ_10321, which encodes a protein with 78% identity with the short-chain alcohol dehydrogenase-like protein Bli-4 of *N. crassa*, strongly induced by light in this fungus [33]. As indicated in the discussion, this gene has sequence similarity to retinol dehydrogenases and could be involved in the metabolism of carotenoids.

**Table 2:** Effect of light and *carS* mutation on the expression of relevant gene groups

Gene (FFUJ_ annotation)	Protein	Fold-change by light	Fold-change in *carS* mutant	Fold-change by light in *carS* mutant
*carS* and structural carotenoid genes				
***carX*** **(11801)**	Carotenoid dioxygenase	96.6	114.7	4.0
***carRA*** **(11802)**	Synthase / cyclase	70.4	208.8	< 2
***carB*** **(11803)**	Desaturase	78.2	160.7	< 2
***carO*** **(11804)**	Rhodopsin	296.8	416.9	3.9
*carT* (07962)	Carotenoid dioxygenase	11.7	35.1	< 2
*carD* (07503)	Aldehyde dehydrogenase	3.7	6.1	< 2
*carS* (08714)	RING finger protein	5.5	7.4	2.5
Other posible carotenoid genes				
(10321)	Putative retinol dehydrogenase	107.5	334.3	2.2
(00634)	Putative retinaldehyde dehydrogenase	−3,9	−3,1	> − 2
Photoreceptor genes				
(00436)	Putative photolyase	35.1	3.7	9.2
*cryD (05732)*	DASH cryptochrome	*57.4*	*12,6*	*6.9*
*vvdA* (06055)	Flavin photoreceptor	87.4	< 2	59.3
*wcoA* (13691)	White Collar flavin photoreceptor	< 2	< 2	< 2
Stress-related genes				
(05128)	Catalase	79.1	133.8	2.1
(11472)	Catalase	54.7	365.1	< 2
(03407)	Catalase	6.7	25.7	< 2
(09119)	Amine oxidase-dehydrogenase	452.5	235.9	2.1
(09320)	Rds1-related	291.4	125.5	4.5
(01993)	Peroxiredoxin subfamily	55.7	35.6	3.0

The genes located in the *car* cluster are in bold

Genes with highest up-regulations by light include those for the photoreceptors Phr, CryD and VvdA (Table 2 and Additional file 3: Table S2A). The effect of light on these genes was formerly known [23, 34, 35], but the influence of CarS on their expression has not been investigated. The *carS* mutation did not affect the transcript levels of the *vvdA* gene and produced moderate stimulations on those of *phr* and *cryD*. In addition, the data confirmed the lack of effect of light on the expression of *wcoA*, which was also not influenced by the *carS* mutation.

**Fig. 4:**
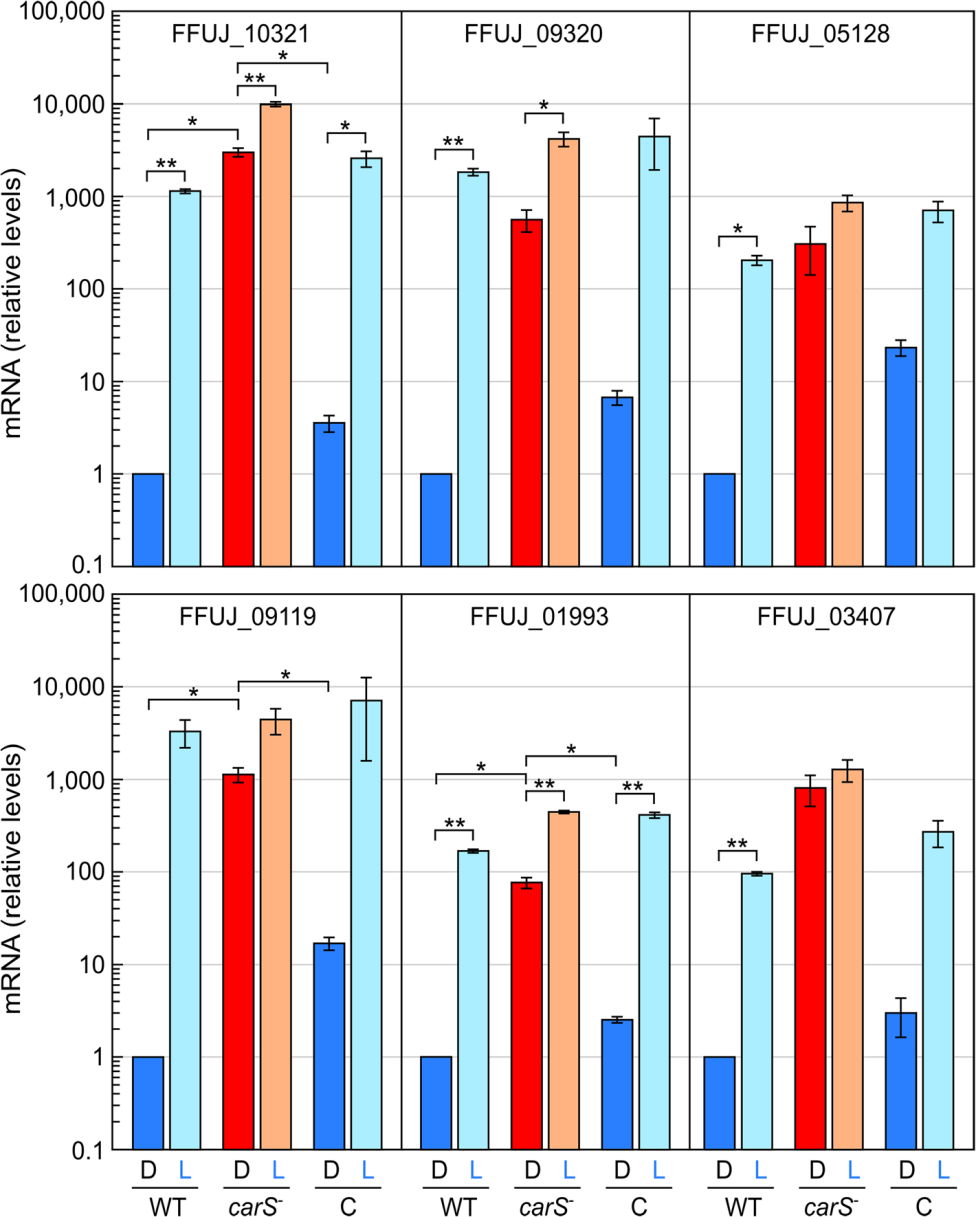
RT-qPCR analysis on the effect of light and *carS* mutation on several putative stress-related genes. RT-qPCRs were carried out with the RNA samples from the wild type (WT), *carS* mutant SG39 (*carS*^*−*^) and complemented strain SG256 (C) used in the RNA-seq study. Darker bars: mycelia grown in the dark (D); paler bars: dark-grown mycelia exposed to light for 1 h (L). Data are referred to the data of the wild strain in the dark and show the mean and error of the mean of the two biological replicates. The unpaired t-test was applied to determine statistical differences between data from dark and light-exposed cultures and between wild type/C and *carS*^*−*^ cultures. Differences found to be statistically significant are indicated with asterisks according to *P* values of 0.0332 (*) and 0.021 (**)

Genes whose expression was strongly regulated by light or by CarS included several genes presumably related to different aspects of cellular responses to stress conditions (Table 2). The third and sixth genes among those with the strongest activations by light (fifth and 23th among those activated by the *carS* mutation) were FFUJ_09119 and FFUJ_O9320 (Additional file 3: Table S2A and C), which encode a FAD flavoprotein and a protein with a ferritin-like domain, respectively. The gene FFUJ_01993, encoding a putative peroxiredoxin, is also in a high position in both tables (positions 27 and 35, respectively). The possible relation of these genes with stress functions is considered in the discussion. The second most activated gene by light is FFUJ_11472 (25th among those activated by the *carS* mutation), which encodes a protein with a catalase domain. Two other genes with catalase domains, FFUJ_05128 and FFUJ_03407, were also remarkably affected by light and by the *carS* mutation (Table 2). For validation purposes, the RNA-seq data for these genes were substantiated essentially by the analyses of mRNA levels by RT-qPCR (representative examples shown in Fig. 4). Due to its possible involvement in the metabolism of carotenoids, the presumed retinol dehydrogenase FFUJ_10321 was also included in the RT-qPCR assays.

### GO term enrichment analysis of activated genes by light and *carS* mutation

The high number of genes whose expression was altered by the *carS* mutation indicates that the function of the CarS protein is not limited to the control of enzymes related to carotenogenesis or stress, but also affects other biological processes in the fungus. To obtain more global information on the role of CarS in *Fusarium* and its relation to light, Gene Ontology (GO) terms were tentatively assigned to the genes that were up-regulated in either of these two conditions. The GO enrichment analysis provides significantly overrepresented GO terms in both sets of differentially expressed genes compared to the background of the genome. The assignment of GO terms was based on the identification of PFAM domains [36]. In the case of the *F. fujikuroi* proteome, at least one significant PFAM domain was found in about 85% of the genes activated by light and 87% of the genes activated in the *carS* mutant.

**Fig. 5:**
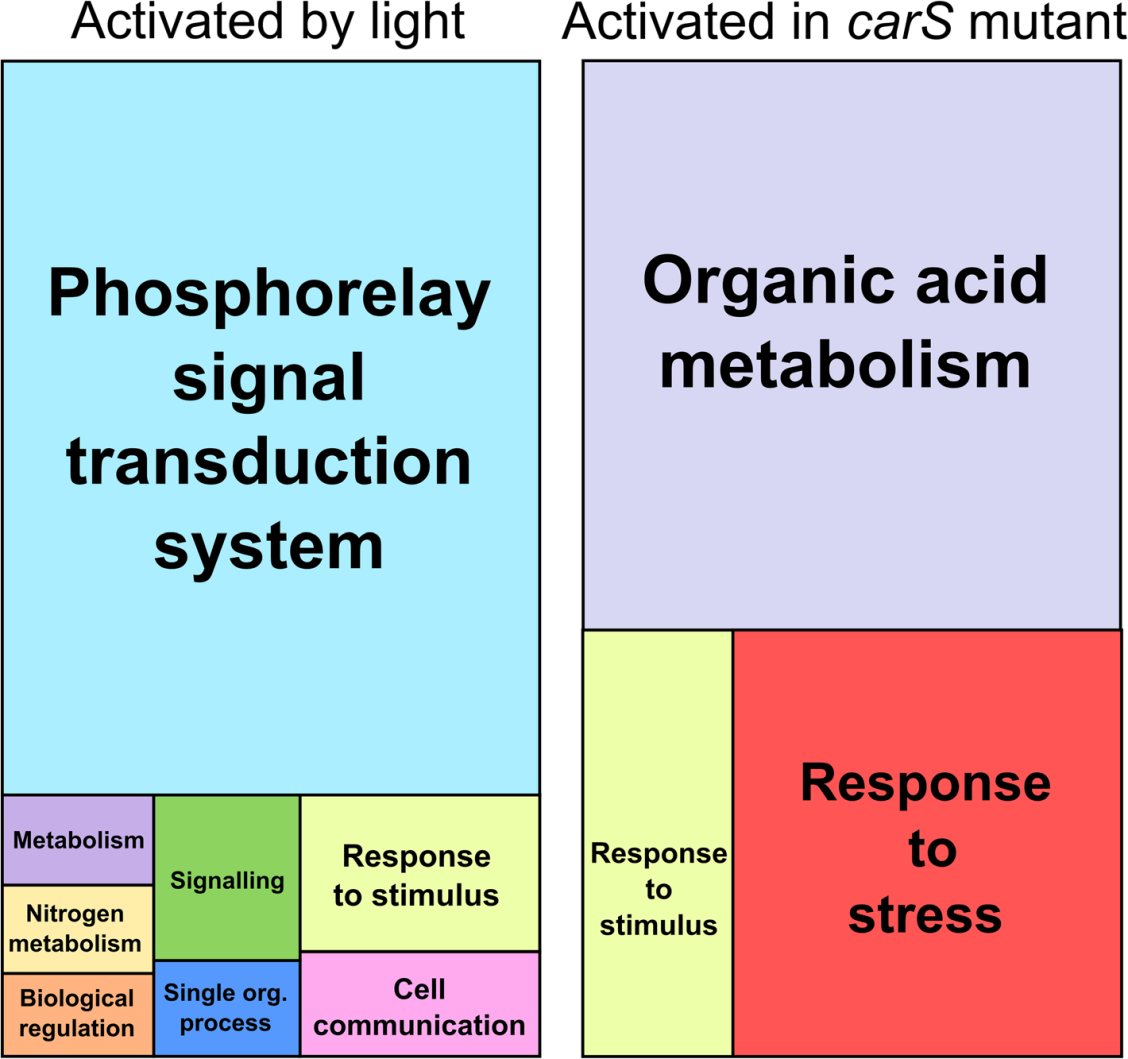
Global effects of light and *carS* mutation in the transcriptome of *F. fujikuroi*. Treemaps representations of Revigo non-redundant GO term enrichment analysis of the *F. fujikuroi* genes induced by light (left, wild type dark vs. wild type light) or by the *carS* mutation (right, wild type dark vs. *carS* mutant dark). Each rectangle area in the treemap represents the −log10 (*p*-value) for the corresponding GO-term

A GO term enrichment analysis performed on this set of genes was summarized with REVIGO [37] and is shown in Fig. 5. The most significant non-redundant GO term for the genes activated by light was “Phosphorelay signal transduction system”, and at a lower extent for other GO terms, some of them also related to signaling or regulation (genes for relevant GO terms listed in Additional file 4: Table S3A). Although activating levels are generally not very high, the appearance of functionally related protein groups is striking. Thus, the list includes six putative histidine kinases, a family of proteins that form two-component systems with partner regulatory proteins. It also stands out the presence in the Metabolic process category of three genes for putative molybdoenzymes of the sulfite oxidase family, which includes nitrate reductase-like enzymes, together with an enzyme involved in the synthesis of molybdopterin, a precursor of the molybdenum cofactor required for the function of these proteins. In this category also appeared the ortholog of the Frequency protein, a central regulator of circadian rhythmicity in *N. crassa* [38].

In the case of the effect of the *carS* mutation (Fig. 5, right panel), the most significant non-redundant GO term was “Organic acid metabolism”, followed by “response to stress”. The first category also includes enzymes supposedly involved in stress, like the aforementioned catalase FFUJ_11472, or the cyanate liase FFUJ_02467, predicted to catalyze the conversion of the toxic cyanate to carbamate, which is spontaneously decomposed to ammonia and carbon dioxide. It is also worth mentioning the presence of three proteins related to DNA damage, the light-activated photolyase Phr (already mentioned in Table 2), a DNA repair enzyme (FFUJ_06571) and a DNA-damage checkpoint protein (FFUJ_13454), as well as some enzymes involved in the metabolism of lipids. In overall, the comparison with the categories of the genes influenced by light indicates that, despite the high coincidence between the genes controlled by light and by CarS, the CarS protein has a narrower range of influence. However, in agreement with the high correlation with the effect of light, 9 of the 17 genes activated by the *carS* mutation (shown in Additional file 4: Table S3) also appear in GO terms for the genes activated by light.

### Effects of light and *carS* mutation on global gene expression in *F. oxysporum*

As an indication of the degree of conservation of the regulations by light and by CarS in *Fusarium*, we extended our study to *F. oxysporum*. In this species, the effect of the *carS* mutation was inferred from the analysis of two independent *carS* mutants, SX2 and SX3 [25]. In this case, to rule out possible effects due to secondary mutations, we only considered as genes influenced by the *carS* mutation those that exhibit at least a 2-fold activation or repression in the two mutant strains. The RNA-seq data and their basic characteristics are described in Additional file 2: Table S1B. The sequence data in relation to the annotated genome identified transcripts for 17,999 genes, with 18,097 isoforms, assignable to 17,687 coding sequences. As was already found for the *F. fujikuroi* data, the representation of the expression values in FKPM with box plot graphs (Additional file 1: Figure S10) showed similar global distributions between all samples, making them comparable. Therefore, as in *F. fujikuroi*, analyses of the data were carried out using the mean of the two replicates as expression value in each strain and experimental condition.

**Fig. 6:**
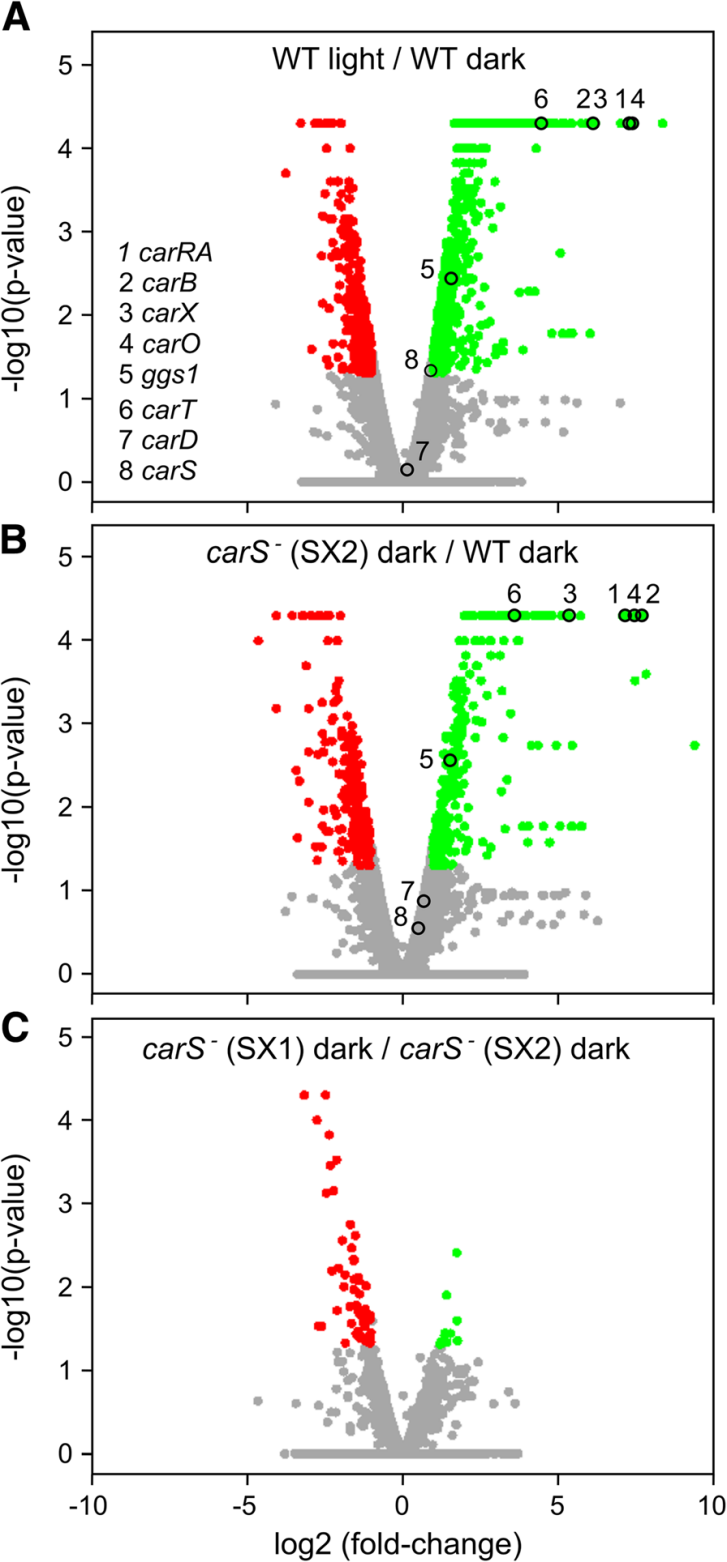
Volcano plot representations of expression data of the *F. oxysporum* genes under different regulatory conditions. **a** Effect of light in the wild type. **b** Effect of the *carS* mutation (*carS* mutant SX2, similar results were obtained with SX1). **c** Comparison of the two *carS* mutants used in the assay. The empty circles indicate the positions of the *car* genes

As already done for *F. fujikuroi* (Fig. 2), the effect of light and *carS* mutation over the *F. oxysporum* transcriptome (gene lists with at least 2-fold changes displayed in Additional file 5: Table S4) was represented in volcano plots (Fig. 6) and scatter plots (Additional file 1: Figure S11). The plots exhibited patterns similar to those of *F. fujikuroi* (compare Fig. 2a with Fig. 6a and Fig. 2b with Fig. 6b), with higher numbers of strongly up-regulated genes (green dots) than down-regulated genes (red dots) as a result of light or *carS* mutation. As in *F. fujikuroi*, the genes of the *car* cluster (dots 1–4 in Fig. 6) exhibited large increases in their transcript levels, accompanied to a lesser extent by the gene *carT* (dot 6), whereas only minor up-regulating effects were observed for *ggs1*, *carD* or *carS* (dots 5, 7 and 8). As predicted, the comparison of the transcriptional patterns between the two *carS* mutants used in the assay revealed minor differences (Fig. 6c), explained by a combination of random variations and the occurrence of different secondary mutations.

### Comparison between the effects of light and *carS* mutation on the *F. oxysporum* and *F. fujikuroi* transcriptomes

**Fig. 7:**
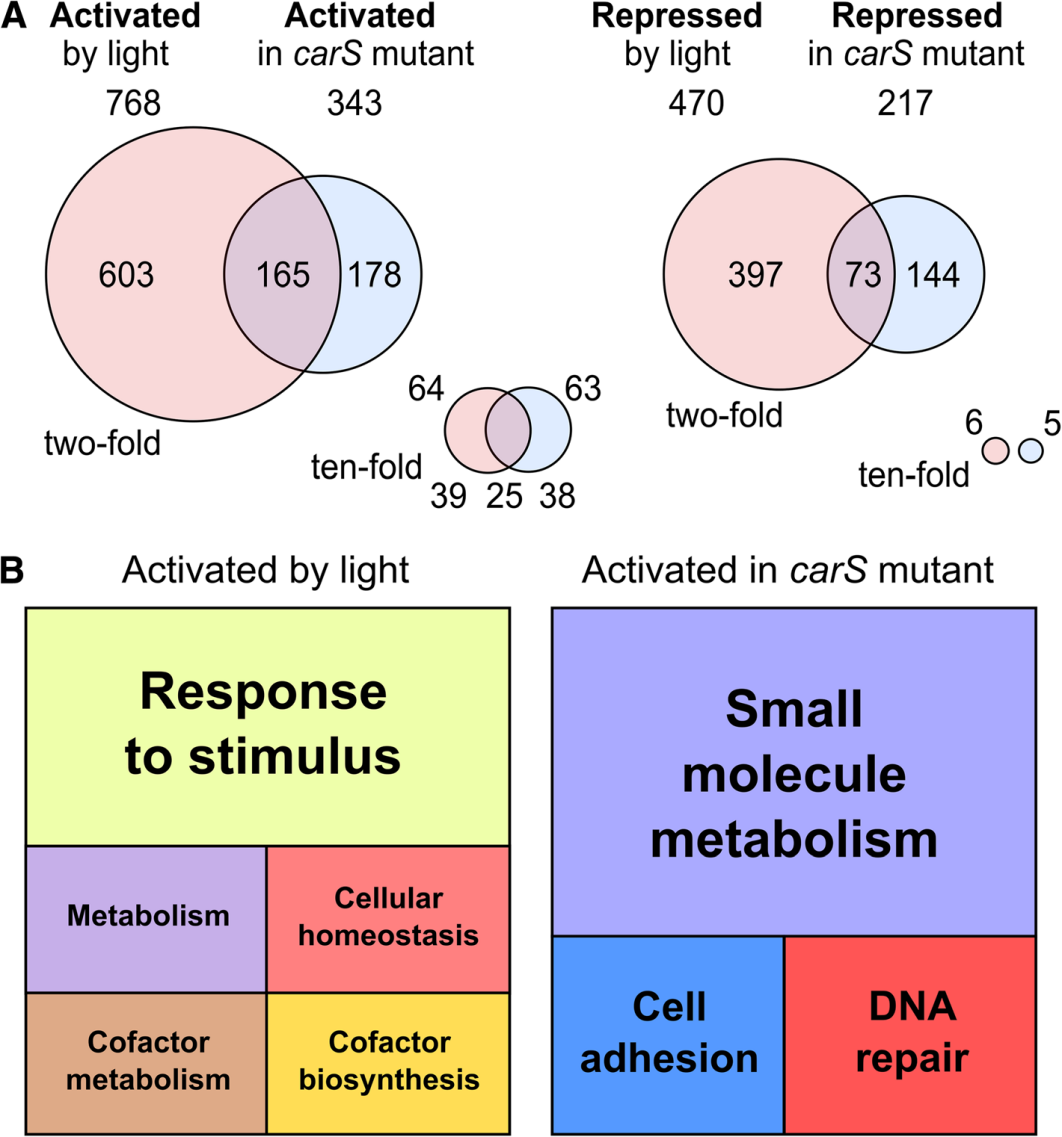
Global effects of light and *carS* mutation in the transcriptome of *F. oxysporum*. **a** Venn diagrams of *F. oxysporum* genes activated or repressed by light or by the *carS* mutation. The data correspond to two-fold and 10-fold thresholds for expression change. The numbers indicate genes corresponding to the conditions indicated above. The surfaces of the circles are proportional to the numbers of genes. The overlapping areas of the circles correspond to genes that coincide in the compared conditions. **b** Treemaps representations of Revigo non-redundant GO term enrichment analysis of the *F. oxysporum* genes induced by light (left) or by the *carS* mutation (right). Each rectangle area in the treemap represents the −log10 (*p*-value) for the corresponding GO-term

The impact of light on the *F. oxysporum* transcriptome was slightly lower compared to *F. fujikuroi*. Using the same 2-fold threshold for modified expression, 768 genes were induced (4.27% of all the genes, compared to 4.79% in *F. fujikuroi*) while only 473 were repressed (2,63%, compared to 3.54%). The effect of the *carS* mutation was also lower in *F. oxysporum*, with 470 induced genes (2.61%, compared to 4.74% in *F. fujikuroi*) and 217 repressed genes (1.21%, compared to 5.44%). The level of overlap between the genes induced by light and by the *carS* mutation (Fig. 7a) was also higher than expected by chance (17.4% for a minimal two-fold increase) but lower than that observed in *F. fujikuroi* (26.9%). The percentage of coincident genes increased also in *F. oxysporum* for higher changes in transcript levels, rising to 24.51% for a ten-fold threshold (Fig. 7a). A significant overlap was also found between genes repressed by light and by the *carS* mutation (11.9%), but no overlap was found between the genes with a decrease of at least ten-fold in their mRNA levels.

The REVIGO summary of the ontology enrichment analysis carried out with the *F. oxysporum* genes activated by light or by the *carS* mutation (Fig. 7b) revealed different GO terms than in *F. fujikuroi*. In this case, the most significant non-redundant GO term for the genes activated by light was “Response to stimulus”, with lower significance levels for other terms, while “Small molecule metabolism” was the most significant among those induced in the *carS* mutant. These differences can be attributed, at least partially, to the lower number of genes in the *F. oxysporum* classifications and to the overlap between the different GO categories. As an indicator of the high similarity between both species related to light and CarS regulation, it was observed that among the genes classified in the GO terms with higher significance, 18 out of the 30 activated genes by light and 5 out of the 7 activated genes in the *carS* mutants in *F. oxysporum* (Additional file 4: Table S3B) were also found in the lists for the GO terms of *F. fujikuroi* (Additional file 4: Table S3A).

**Fig. 8:**
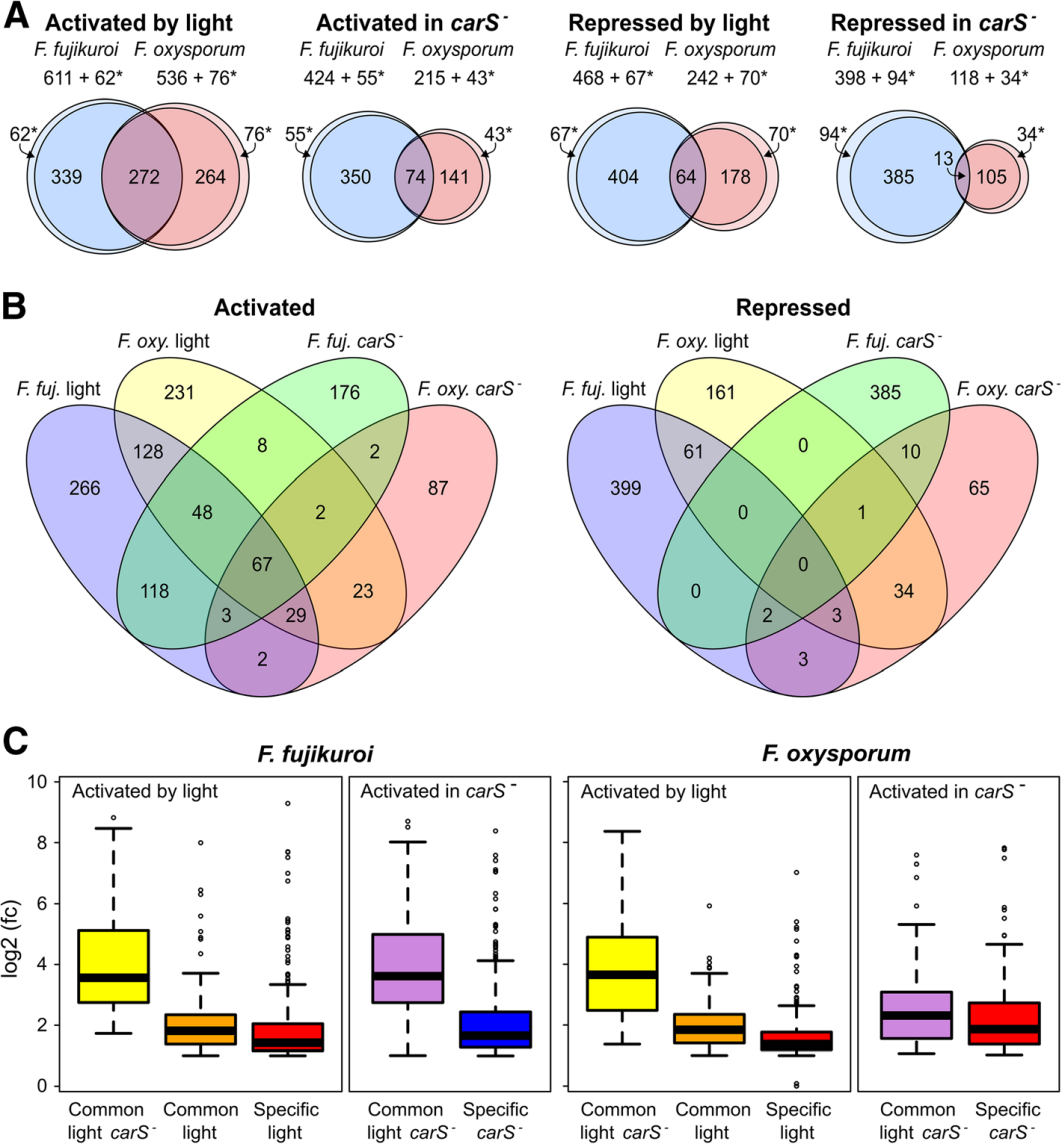
Overlaps between *F. fujikuroi* and *F. oxysporum* shared genes activated or repressed by light or by the *carS* mutation. **a** Overlaps between activated or repressed genes by light or by *carS* mutation between *F. oxysporum* and *F. oxysporum*. Genes without orthologs in the other species are indicated with an asterisk. The overlap between the genes activated by light in *F. fujikuroi* and *F. oxysporum* is significant, with an odds ratio enrichment of 9.15 and a *p*-value < 2.2 × 10^− 16^. The overlap between the genes repressed by light in *F. fujikuroi* and *F. oxysporum* is also significant with, an odds ratio enrichment of 6.27 and a *p*-value < 2.2 × 10^− 16^ (*p* values computed with the Fisher’s exact test). **b** Representation of overlaps between the Venn diagrams shown above. Only genes with orthologs in the *F. fujikuroi* (*F. fuj.*) and *F. oxysporum* (*F. oxy.*) genomes are considered. **c** Box-plot representations of the fold changes in the expression of genes simultaneously activated by light and *carS* mutation (common light *carS*^*−*^) or only by light (common light) in *F. fujikuroi* and *F. oxysporum* compared to those activated specifically in only one of the species (specific light and specific *carS*^*−*^). There were only three genes activated only by the *carS* mutation in both *Fusarium* species (common *carS*^*−*^), and therefore the box-plot is not represented

A comparison using the Bidirectional Best Hit algorithm [39] between the genomes of the two *Fusarium* species revealed 11,918 genes with possible orthologs in both strains (Additional file 6: Table S5), which will be referred to hereafter as common genes, while 3177 (21.0%) and 6081 (33.8%) genes were specific to the *F. fujikuroi* and *F. oxysporum* genomes, respectively. Light had less influence on the specific genes in both species; thus, of the sets of 3177 and 6081 genes, only 9.2 and 12.4% were activated by light and 11.5 and 16.7% were activated by the *carS* mutation, respectively (Fig. 8a). In relation to the common genes, when comparing the sets of genes activated by light in both species, we found 272 coincidences, whereas the number of genes coinciding among those repressed by light was only 64. The coincidence in the genes activated by the *carS* mutation was lower (74, Fig. 8a), suggesting differences in the regulatory roles played by the CarS protein in both *Fusarium* species. However, again the number of matches was greater for induction than for repression in the *carS* mutants.

An overall representation of the matches between the genes regulated by light and CarS in *F. fujikuroi* and *F. oxysporum* in a Venn diagram revealed a greater degree of overlap for the activated genes than for the repressed genes (Fig. 8b). If the focus is on the 272 genes activated by light and the 74 genes activated in the *carS* mutant in both species, we found 67 genes that are activated in both regulatory conditions; however, no coincidences were found between the 64 genes repressed by light and the 13 genes repressed by the *carS* mutation. The relative changes in mRNA levels for the 67 genes were greater in the different species and regulatory conditions than in the genes that coincide only in the activation by light or by the *carS* mutation (Fig. 8c). This set of genes (Additional file 7: Table S6) includes the four genes of the *car* cluster, and the genes *carT* and *ggs1*, as well as the genes for the cryptochromes CryD and FFUJ_03105/FOXG_02060, and different enzymes related to stress or other aspects of cellular metabolism.

## Discussion

RNA-seq technology is a powerful tool that allows the quantification of mRNA levels of all genes in the genome of an organism. We have used this methodology to investigate the impact on the global transcriptome of two regulatory conditions that control carotenogenesis in *Fusarium*: up-regulation by light and down-regulation by the RING-finger protein CarS. In addition, we have used the relationships between both regulatory effects to explore their possible regulatory connections. The effect of light has been investigated in other species, but this is the first report on a transcriptomic analysis on the function of a RING-finger protein in fungi. Our RNA-seq data showed that illumination for 1 hour changed at least two-fold the expression of about 8% of the *Fusarium* genes in our experimental conditions. This is a smaller impact than that observed in *N. crassa* [40], a major reference in studies of fungal photobiology. In this fungus*,* the number of genes whose expression changed at least two-fold by light reached 31% of those expressed. The analyses in *N. crassa* were done with four times of illumination, 15 min, 1 h, 2 h, and 4 h, what could explain at least in part the 4-fold difference in the number of affected genes between both species. A more detailed time course of light exposure could increase the number of affected genes in *Fusarium*.

The impact of the mutation of the *carS* gene on the *Fusarium* transcriptome is quantitatively not very different from that of light, with about 10% of the genes affected directly or indirectly using the same fold change threshold. In this case, comparisons with *N. crassa* are not possible, because *carS*-like mutants have not been described in this fungus and there is no clear *carS* ortholog in its genome. Either after illumination or as a cause of the *carS* mutation, activation predominates among the main effects in *Fusarium* expression. It seems very likely that the effects of light and *carS* mutation share control elements, as indicated by the significant overlap between the affected genes in both regulatory conditions. This conclusion is reinforced by the drastic decrease in the genes regulated by light in the *carS* mutant.

In a previous screening of genes regulated by the CarS protein, based on a subtractive hybridization protocol, only about 60 genes were identified [31]. This group, which included genes activated or repressed in a *carS* mutant, represented less than 0.5% of the annotated *F. fujikuroi* genes, which corresponded to an experimental efficiency at least an order of magnitude lower than that obtained with the RNA-seq method. This difference suggests that the data of subtractive hybridization are biased towards those genes with higher expression. In addition, in contrast to the efficiency of the RNA-seq technique, the subtractive hybridization study did not reach saturation, as revealed by the absence of some of the genes of the *car* cluster in the scrutiny. However, the present RNA-seq analysis confirmed some of the genes identified with this previous approach. These include the homologs of the *rds1* and *bli-4* genes. The case of the *bli-4* homolog*,* FFUJ_10321*,* is particularly interesting. This 341-aa protein exhibits a significant similarity with retinol dehydrogenases (RDHs) of mammals, as RDH11, RDH12, RDH13, and RDH14 of *Mus musculus* (e.g., 31% identity along 301 residues of the 316 residues of RDH12 [Q8BYK4.1], *E*-value 6e-18). RDHs catalyze the conversions between retinol and retinal, and in *F. fujikuroi* such enzymatic activity could be related with the retinal produced by the CarX enzyme [41]. RDH13 (Q8NBN7.2, 31% identity with FFUJ_10321 along 210 residues) has received special attention among mammalian RDHs. RDH13 is located on the outside of the inner membrane of the mitochondria in the pigmented epithelium of the retina, where it presumably protects against oxidative damage caused by light [42]. In humans, RDH13 is also found in mitochondria and could be involved in protection against oxidative stress caused by retinaldehyde. There is evidence that RDH13 specifically protects against light-induced apoptosis in photoreceptors and, therefore, inhibits cell death associated with mitochondria.

Our RNA-seq analyses point to the *car* cluster as one of the main regulatory targets of the CarS protein. In fact, the genes of this cluster are among the most influenced by light. However, other genes strongly regulated by light, as those of the photoreceptors CryD and VvdA, are hardly affected by the *carS* mutation compared to light, providing examples of the separation of both regulatory mechanisms. CryD and VvdA play roles in the regulation of carotenogenesis by light [21] and the photoinduction of the *cryD* and *vvdA* genes was described previously [23, 35]. On the contrary, neither the light nor the *carS* mutation affect the expression of the gene for the main photoreceptor involved in the photoinduction of carotenoids, the WC-1 protein WcoA. The lack of effect of the *carS* mutation on the levels of *wcoA* mRNA does not rule out a possible role of CarS in the control of WcoA function, e.g., at the level of ubiquitination as described for its homolog CrgA on the Wc-1 protein Mcw-1 in *M. circinelloides* [32]. WcoA is the main transcriptional inducer of the genes of the *car* cluster, either in light or in darkness [21], so we may anticipate some type of regulatory connection with CarS, which has not been yet clarified.

The analysis of alterations in the transcriptome as a result of illumination or the absence of CarS in the dark reveals large changes in mRNA levels of several genes related to stress functions (Table 2). FFUJ_09119 encodes a FAD flavoprotein belonging to the family of pyridine nucleotide-disulphide oxidoreductases, a group that includes glutathione reductase, trypanothione reductase, lipoamide dehydrogenase, mercuric reductase, thioredoxin reductase, and alkyl hydroperoxide reductase. FFUJ_09320 contains a ferritin-like domain (*E*-value 1.70e-21) and it is homologous to the transcription factor Rds1, with a function in stress-related responses in *Schizosaccharomyces pombe* [43] and related to drug sensitivity in *Saccharomyces cerevisiae* [44]. FFUJ_01993 encodes a protein with an OsmC super family domain (5.83e-41), related to osmotic stress. This family also includes an organic hydroperoxide detoxification protein. Three other proteins strongly regulated by light and CarS contain catalase domains, and therefore could play roles in the defense against oxidative stress. FFUJ_11472 encodes a large protein consisting of a carboxy sulfite oxidase domain (*E*-value 2.88e-113) followed by a catalase domain (*E*-value 0). FFUJ_05128 has a catalase domain that covers most of the protein (*E*-value 0) and is very similar to a putative peroxisomal catalase from the yeast *Candida utilis* [45], with 54% identity along a stretch of 483 of the 577 residues of the protein. The last one is FFUJ_03407, a small protein (229 residues) with a putative catalase domain segment (residues 61 to 138, 2.40e-29). Light or CarS exerted a lesser influence on other predicted catalase genes of *F. fujikuroi*. Thus, FFUJ_03451 and FFUJ_11706, orthologs of the *cat-1* and *cat-2* catalase genes of *N. crassa* [46], were stimulated 3.2 and 2.1 fold by light but were not significantly influenced by the *carS* mutation.

Mitochondria are a primary target of H_2_O_2_ damage in fungi [47]. This could explain why some of the genes that are induced to a greater degree by light or *carS* mutation may be involved in the prevention of stress in the mitochondria. Interestingly, carotenoids, especially xanthophylls, have been reported as antioxidant agents in fungi [48]. In fact, different indirect data suggest that neurosporaxanthin has antioxidant activity. In *N. crassa*, the synthesis of this xanthophyll in response to light is greater in the absence of superoxide dismutase activity [49]. In *F. aquaeductuum*, the presence of H_2_O_2_ in the culture medium stimulates the synthesis of neurosporaxanthin in the dark [12]. Considering the presumed antioxidant properties of carotenoids, the coincidence in the regulation of the expression of carotenogenesis genes with some genes related to oxidative stress is very suggestive.

The GO analyses have revealed the participation of light and CarS in the control of some genes for proteins of potential interest in signaling regulations, such as six genes for predicted histidine kinases, also found under the GO term “Biological regulation” (Additional file 4: Table S3). These proteins participate in two-component systems that are very abundant in prokaryotes, but are also found in lower eukaryotes, where they participate in different signaling pathways [50]. Five of these genes exhibit modest activations by light, usually ca. 2–3-fold, but the putative histidine kinase gene FFUJ_10367 stands out with 33-fold activation under light. Two of them, FFUJ_02424 and FFUJ_13427, are related with the Nik-1 (Os-1) protein of *N. crassa*, involved in osmotic sensing. However, they exhibit a lower similarity with Nik-1 (17 and 19% identity) than the real Nik-1 ortholog, FFUJ_02727 (80% identity), and therefore they are allegedly involved in other unknown signal transduction mechanism. This is also true for the other putative histidine kinase genes, whose lack of sufficient similarity to functionally characterized orthologs in other organisms hinders the deduction on their putative regulatory functions. In the category of metabolic process, the presence of three putative genes of the nitrate reductase group stands out. In contrast to these genes, the assimilatory nitrate reductase gene FFUJ_12277, *niaD* (52,9% identity with *niaD* of *A. nidulans*) is repressed by light (3,5-fold reduction, Additional file 3: Table S2B). The occurrence of more than one nitrate reductase can be explained by their specialized participation in other metabolic processes, such as denitrification or ammonia fermentation, related with hypoxia and already described in *F. oxysporum* [51].

The *Fusarium* species, highly ubiquitous in nature, usually infect a limited range of plant species and possess specific virulence genes. A comparative study between the genomes of *Fusarium graminearum*, *Fusarium verticillioides* and *F. oxysporum* f. sp. Lycopersici revealed the existence of large lineage-specific genomic regions, in some cases covering whole chromosomes, that in *F. oxysporum* reach up to one quarter of the genome [52]. The existence of these species-distinct genomic regions, presumably involved in their host-specific pathogenicity, explains the high number of non-coincident genes between *F. oxysporum* and *F. fujikuroi*. Interestingly, species-specific genes were less affected by light and by CarS than shared genes, suggesting regulatory functions that precede pathogenic specializations in the *Fusarium* group.

In this work, we have used *F. fujikuroi* and *F. oxysporum* to evaluate the degree of conservation in different *Fusarium* species of transcriptional regulation by light and by CarS. The reason for choosing these species is the availability of *carS* mutants [25], not yet described in other *Fusarium* species. The comparison of the effect of light and the *carS* mutation in *F. fujikuroi* and *F. oxysporum* revealed a set of 67 genes activated by both regulatory conditions in both species, of which ca. 20% are uncharacterized proteins. The group of 67 genes exhibits higher transcriptional changes that in average and should be therefore particularly informative on the regulatory relations between the controls by light and the CarS protein in these fungi. With the exception of *carD*, all the structural genes associated to carotenoid metabolism are found in this group, confirming the major role of light and CarS in the regulation of carotenoid biosynthesis, possibly associated with the formation of photoactive CarO rhodopsin. The rest of the proteins are involved in very diverse functions, related to stress, signaling, and carbohydrate or lipid metabolism. The function of the uncharacterized proteins, or the occurrence of possible functional links between the identified proteins, should be subjects of future research.

## Conclusions

Light and the loss of function of the *carS* gene exert mainly activating effects on the transcriptome of *Fusarium fujikuroi*, with a greater functional diversity in the case of genes influenced by light. The number of the latter decreases drastically in the *carS* mutant, indicating that CarS modulates the expression of many genes regulated by light. Approximately 27% of genes activated at least twice by light or by the *carS* mutation are coincident, raising this percentage to 40% for higher activation thresholds. The large overlap between the two sets of genes confirms regulatory connections between the control of gene expression by light and the CarS protein, which seems to play a role by keeping the mRNA levels of many light-inducible genes low in the dark. According to the magnitude of transcriptional changes, the genes for carotenogenesis are major regulatory targets of light and CarS. Other common regulatory targets are proteins with putative connections with stress responses. They include several genes with catalase domains, which is consistent with roles of light and CarS protein in the control of oxidative stress. Some genes that are strongly induced by light are not affected by the *carS* mutation, indicating separate mechanisms of action. In this group the genes for the photoreceptors CryD and VvdA, that play a role in the regulation of carotenogenesis, stand out. The effects of light and CarS mutation were also investigated in *F. oxysporum,* that exhibited a similar global response than *F. fujikuroi*, but with quantitative and qualitative differences in their respective sets of affected genes. However, 67 genes exhibited a remarkable up-regulation by light and by the *carS* mutation in both species, indicating the conservation of regulatory roles.

## Methods

### Strains and culture conditions

The strains of *Fusarium fujikuroi* were wild type IMI58289, obtained from the Imperial Mycological Institute (Kew, Surrey, England), the *carS* mutant SG39 [24] and the complemented strain SG256, isolated from a sectoring transformant obtained by introduction of the wild type *carS* gene in the mutant SG39 [31] (Additional file 1: Figure S2). The strains of *Fusarium oxysporum* were wild type f. sp. *lycopersici* 4287 (race 2), kindly provided A. Di Pietro (Universidad de Córdoba, Spain) and the 4287-derived *carS* mutants SX1 and SX2 [25].

Strains were cultured in DG minimal medium [composition per liter: 30 g glucose, 3 g NaNO_3_, 1 g KH_2_PO_4_, 0.5 g KCl, 0.5 g MgSO_4_·7H_2_O and microelements, [53]] or in DGasn medium (the same as DG medium but with 3 g asparagine instead of NO_3_Na). To obtain conidia of the strains of *F. fujikuroi*, they were grown on EG medium (composition per liter: 1 g glucose, 1 g yeast extract, 1 g NO_3_NH_4_, 1 g KH_2_PO_4_, 0.5 g MgSO_4_·7H_2_O, 16 g agar).

For RNA-seq or RT-qPCR analyses, 10^6^ conidia were inoculated in 100 ml of DG medium in 500-ml Erlenmeyer flasks and incubated in the dark for 3 days in an orbital shaker. Subsequently, 25 ml samples of the cultures were transferred to Petri dishes under red safelight and incubated for 1 hour in the dark or under white light. Illumination was provided with a set of four fluorescent tubes (Philips TL-D 18 W/840) at a distance of ca. 60 cm, yielding a light intensity of 7 W m^− 2^. When indicated, shorter light pulses were given followed by a subsequent incubation in the dark for up to 1 hour. The process with *F. oxysporum* was the same, except that the 3-day cultures were incubated in 145-mm Petri dishes with 80 ml of DGasn medium. Mycelia samples were obtained by filtration, frozen immediately in liquid nitrogen, and stored at − 80 °C.

### Expression analysis

The transcript levels of the investigated genes were analyzed by reverse transcription qPCR (RT-qPCR), achieved according to MIQE guidelines [54]. Relevant RT-qPCR methodological details not mentioned in this section are described in Additional file 8. Total RNA samples were extracted from 150 to 200 mg of ground mycelia and treated with DNAse with the NucleoSpin RNA kit (Macherey-Nagel, Düren, Germany). In the case of the effect of light pulse duration (data in Fig. 1), RNA was extracted with the RNeasy RNA isolation kit (Qiagen, Chatsworth, CA, USA). In the case of the effect of light and *carS* mutation (data in Fig. 4), the extraction procedure is described in the RNA-seq section. RNA concentrations were estimated with a Nanodrop ND-1000 spectrophotometer (Nanodrop Technologies, Wilmington, DE, USA). Concentration and A260/A280 absorbance ratios of the RNA samples are indicated in Additional file 8. Samples of 2.5 μg of RNA were retrotranscribed to cDNA with Transcriptor first-strand cDNA synthesis kit (Roche, Mannheim, Germany), and final cDNA concentrations were adjusted to 25 ng μl^− 1^. RT-qPCR analyses were performed in a LightCycler 480 real-time instrument (Roche). For amplification and detection, LightCycler 480 SYBR Green I Master (Roche) was used following manufacturer reaction protocol. Genes and primer sets (forward vs reverse in 5′- > 3′ orientation and amplicon length) were *carRA* (CAGAAGCTGTTCCCGAAGACA vs TGCGATGCCCATTTCTTGA, 65 bp), *carB* (TCGGTGTCGAGTACCGTCTCT vs TGCCTTGCCGGTTGCTT, 68 bp), FFUJ_10321 (GCTCGCAACCCGTCAATT vs ACGGCGACTTTGTTGATTAGGT, 60 bp), FFUJ_09320 (CAACGATGAGGACCGCTTTC vs AGTCGCGTGGCCGATTT, 61 bp), FFUJ_05128 (GCGCCTTCAGCAACAGACA vs TGACGGTCTGATTCGTAGGATTG, 74 bp), FFUJ_09119 (CCCGGAACCGTTCAATACTG vs TTATCTCCGTTCTGAGCCATCA, 66 bp), FFUJ_01993 (AATCCATCAACCCTCCTCAGATC vs TCTTTGTTCTGCTGGGTGCTT, 68 bp) and FFUJ_03407 (CTCCCAACAGCTTCGCTTACA vs TGTTGTCGCTGACCTGATACG, 69 bp). Coding sequences and amplicon locations are indicated in Additional file 9. Transcript levels for each gene were normalized against the tubulin beta chain gene FFUJ_04397 (CCGGTGCTGGAAACAACTG vs CGAGGACCTGGTCGACAAGT, 69 bp) and the glyceraldehyde 3-phosphate dehydrogenase (GAPDH) gene FFUJ_13490 (GTGACCTCAAGGGCGTTCTG vs CGAAGATGGAGTTTGTGTT, 84 bp). FFUJ_04397 was used as a reference gene for constitutive expression in former RT-qPCR studies in *Fusarium fujikuroi* (see, e.g., [19, 21, 23, 28, 35]), and the GAPDH gene was recently used as an internal control in RT-qPCR studies in *F. graminearum* [55, 56].

Statistical differences between selected RT-qPCR data in Fig. 4 were analyzed with the Student’s t-test (unpaired t-test) with the GraphPad Prism 7.0 software (http://www.graphpad.com), using *P* values of 0.0332 (*), 0.021 (**) and 0.002 (***).

### RNA-seq procedures

Total RNA samples were extracted with Trizol (Invitrogen, Paisley, UK) using the protocol described by the manufacturer. The samples were processed with the Illumina protocol by the company Life Sequencing (Valencia, Spain), which consists of the following steps: (1) enrichment in poly-A RNA to discard ribosomal RNA or other non-coding RNAs; (2) fragmentation of RNA into pieces of 100 to 300 bp; (3) synthesis of double strand cDNA by reverse transcription with oligo dT; (4) addition of adapters to terminal ends; and (5) Selection of fragments of appropriate size and subsequent amplification by PCR. Sequencing was achieved with the Illumina HiSeq Platform in 50SE composition using the Single-End methodology.

For statistical and graphic analyses of the sequence data, we used the R programming language, based on our own scripts, and the Bioconductor data analysis libraries (http://www.bioconductor.org). The Integrative Genomics Viewer IGV application (IGV) version 2.3.57 was used for mapping visualization [57].

### Bioinformatics analyses

The processing of RNA-seq sequencing data was carried out through the high-performance computing facilities provided by the CICA platform (Centro Informático Científico de Andalucía, Sevilla, Spain) using the Tuxedo protocol. For quality control, the software package FastQC was used. For short read mapping to reference genomes, transcript assembly and differential expression, the software tools Bowtie, TopHat, Cufflinks, Cuffmerge and Cuffdiff [58] were used with default parameters. Tophat was used to map short reads to the reference genome and Cufflinks to assemble sample specific transcripts. The results obtained with Cufflinks were integrated with the Cuffmerge tool to generate the complete transcriptome associated with our study. Gene expression levels were measured as FPKM (fragments per kilobase of exon per million of mapped reads) with the Cuffdiff tool, which normalizes the expression levels obtained in the sequencing eliminating biases due to differences in the length of the transcript and the size of the library [59]. The rest of the analysis and the visualization of the results were done with the CummeRbund package, obtained from the Bioconductor platform (https://www.bioconductor.org), which manages the files through R scripts.

To verify the quality of the sequencing data, the dispersion and squared coefficient variation (SCV) of the samples were analyzed. No substantial overdispersion was observed and SCV did not indicate a high degree of variability between the replicates. The differentially expressed genes were selected based on criteria combining a log2 fold change of 1 and a *p*-value of 0.05.

GO categories were generated for the genomes of *F. fujikuroi* and *F. oxysporum* by detecting protein domains using the PFAM database and applying the available pfam2GO correspondence. This allowed the annotation of approximately 50% of the genes. GO term enrichment analysis was performed using the Bioconductor R package topGO with a p-value of 0.05 computed by Fisher’s exact test. REVIGO (http://revigo.irb.hr) was used to eliminate the redundancy from enriched GO terms based on a measure of semantic similarity and visualize the results as treemaps [37].

## Additional files

Additional file 1: **Figure S1.** Biochemical reactions and enzymes involved in the metabolism of carotenoids in *Fusarium*. The arrowheads indicate the reaction sites in the molecules. The genomic organization of the structural genes is shown in the box. **Figure S2.** Molecular steps in the generation of the complemented strain SG256. The *carS* mutant SG39 was obtained by chemical mutagenesis. In a first complementation step, a plasmid containing the wild *carS* allele was introduced at the *carS locus* of SG39 by homologous recombination. This strain is unstable due to spontaneous loss of the plasmid by new recombination events. The stable complemented strain SG256 was obtained by the loss of the plasmid carrying the *carS* mutant allele. **Figure S3.** Quality verification according to different parameters shown through the FastQC program for a representative sample (wild type dark 1). A. Quality representation according to the position of the nucleotide for all the readings detected in the sequencing. Our analysis corresponds to fragments about 50 bases in length, in which no appreciable quality problems were detected throughout the readings. The blue line represents the mean values and the red line the median. Practically all the readings are in the green zone, which confirms its quality. B. Measurement of the average quality of each reading. The quality is represented in abscissa and the number of readings in ordinates. It can be seen that the average quality is higher than 30. Sequences with a more irregular distribution and values ​​below 20 would indicate quality problems. C. Distribution of the base content throughout the readings. There is a uniform distribution, except at the beginning of the readings, a deviation attributed to the elimination of the 5′ adapters. This is considered normal in the method and does not alter the results. The G + C content remains constant and close to the previously detected value of 50%. D. Distribution of sequence lengths. Most of the readings were around 50 bases. **Figure S4.** Boxplot representation of the samples of *F. fujikuroi* used in the study. The boxes represent 50% of the variation (±1 quartile). The lines in the boxes indicate the mean. Points indicate the farthest genes from the mean, either for activation (positive values for the Y axis) or for repression (negative values for the Y axis). **Figure S5.** Global distribution of the repetition of gene expression between biological replicates in function of the values of gene expression. The internal lines represent the average variation. CV: Variation coefficient, used as a measure of the dispersion of expression values. **Figure S6.** Scatter plot representations of the effect of light and SG39 genotype (*carS*) on transcript levels of the *F. fujikuroi* genes. The numbers indicate genes related to carotenogenesis according to the legend shown in panel WT light / WT dark. **Figure S7**. Graphic representation of the expression changes for the 30 genes with the higher activation (ascending arrowheads) and repression (descending arrowheads) as a result of light (blue symbols) or *carS* mutation (red symbols) in *F. fujikuroi* (A) and *F. oxysporum* (B). Genes were ordered according to increasing expression fold-changes. The genes of *F. fujikuroi* are shown in grey in panel B for a better comparison. The sets of 30 genes exclude one gene from *F. fujikuroi* and nine genes from *F. oxysporum* without detectable expression in the wild type in the dark, resulting in infinite fold change (indicated as [#∞] in a box in the top of their corresponding groups). The genes of the *car* cluster are indicated with circles and numbers according to the inner legends. In the case of induction by light in *F. oxysporum*, *carT* was also found in the represented genes. **Figure S8.** Recovery of wild expression pattern in the complemented strain SG256. Above: Venn diagrams representing the overlap between genes activated or repressed in SG256 in relation to the *carS* mutant SG39 in the dark. Below: Venn diagrams of matches between the genes activated and repressed by light in the wild strain and in SG256. The number of coincident genes is shown in the overlapping areas. The surfaces of the circles and the intersections are proportional to the number of genes. **Figure S9.** Venn diagrams of *F. fujikuroi* genes with opposite effects by light or by the *carS* mutation. **A-B** Venn diagrams of *F. fujikuroi* genes activated or repressed by light or by the *carS* mutation. The effect of the *carS* mutation was corrected with the data of the SG256 complemented strain. **C-D** Venn diagrams of *F. fujikuroi* genes activated or repressed by light in the wild type or in the *carS* mutant SG39. In all the diagrams, the numbers indicate genes that correspond to the conditions mentioned above. The surfaces of the circles are proportional to the numbers of genes. The intersections between the circles in diagrams A and B correspond to genes coinciding in the conditions compared. The set of genes repressed/activated by the *carS* mutation in dark conditions did not significantly overlap with the sets of genes activated/repressed in the wild type (WT) according to *p*-values of 0.49 (22 genes in diagram A) and 0.86 (11 genes in diagram B), respectively. There was no overlap in the sets of genes shown in diagrams C and D. **Figure S10.** Boxplot representation of the samples of *F. oxysporum* used in the study. The boxes represent 50% of the variation (±1 quartile). The lines in the boxes indicate the mean. Points indicate the farthest genes from the mean, either for activation (positive values for the Y axis) or for repression (negative values for the Y axis). **Figure S11.** Scatter plot representations of the effect of light and *carS* mutation on the transcript levels of the *F. oxysporum* genes. The numbers indicate genes related to carotenogenesis according to the legend shown in panel SX1 dark / SX2 dark. (PDF 4044 kb)

Additional file 2: **Table S1.** Basic features of the sequences used in the RNA-seq analysis described in this work. (PDF 26 kb)

Additional file 3: **Table S2.** List of *F. fujikuroi* genes influenced at least two-fold by light (A and B) or by the *carS* mutation (C and D) sorted by their degree of activation (Tables S2A and S2C) or repression (B and D). (XLSX 150 kb)

Additional file 4: **Table S3.** GO term enrichment in genes activated by light or by the *carS* mutation in *F. fujikuroi* (A) and *F. oxysporum* (B). (PDF 128 kb)

Additional file 5: **Table S4.** List of *F. oxysporum* genes influenced at least two-fold by light (Tables S4A and S4B) or by the *carS* mutation (C and D) sorted by their degree of activation (A and C) or repression (B and D). (XLSX 93 kb)

Additional file 6: **Table S5.** Identification of *F. fujikuroi* and *F. oxysporum* potential orthologs using the Best Bidirectional Hit algorithm. (XLSX 417 kb)

Additional file 7: **Table S6.** Orthologous genes activated by light and *carS* mutation in *F. fujikuroi* and *F. oxysporum.* (XLSX 38 kb)

Additional file 8: **Tables S7** and **S8**, **Figures S12** and **S13.** Methodological information of the RT-qPCR procedure. (PDF 672 kb)

Additional file 9: Sequences of the genes used in the RT-qPCR experiments and amplicon locations. (PDF 68 kb)

## Abbreviations

CICA: Centro Informático Científico de Andalucía
FPKM: Fragments per kilobase of exon per million of mapped reads
GO: Gene ontology
IGV: Integrative Genomics Viewer
RDHs: Retinol dehydrogenases
RNA-seq: Ribonucleic acid sequencing
RT-qPCR: Quantitative reverse transcription PCR
SCV: Squared coefficient variation
WC: White Collar
WCC: White Collar complex
WT: Wild type
