# Supplementary material for: Comparative transcriptomic analysis unveils interactions between the regulatory CarS protein and light response in Fusarium
Source: BMC Genomics. 2019 Jan 21;20:67. doi: 10.1186/s12864-019-5430-x (PMC6340186; doi:10.1186/s12864-019-5430-x)
Supplement: Supplementary file 1 — Figure S1. Biochemical reactions and enzymes involved in the metabolism of carotenoids in Fusarium. The arrowheads indicate the reaction sites in the molecules. The genomic organization of the structural genes is shown in the box. Figure S2. Molecular steps in the generation of the complemented strain SG256. The carS mutant SG39 was obtained by chemical mutagenesis. In a first complementation step, a plasmid containing the wild carS allele was introduced at the carS locus of SG39 by homologous recombination. This strain is unstable due to spontaneous loss of the plasmid by new recombination events. The stable complemented strain SG256 was obtained by the loss of the plasmid carrying the carS mutant allele. Figure S3. Quality verification according to different parameters shown through the FastQC program for a representative sample (wild type dark 1). A. Quality representation according to the position of the nucleotide for all the readings detected in the sequencing. Our analysis corresponds to fragments about 50 bases in length, in which no appreciable quality problems were detected throughout the readings. The blue line represents the mean values and the red line the median. Practically all the readings are in the green zone, which confirms its quality. B. Measurement of the average quality of each reading. The quality is represented in abscissa and the number of readings in ordinates. It can be seen that the average quality is higher than 30. Sequences with a more irregular distribution and values ​​below 20 would indicate quality problems. C. Distribution of the base content throughout the readings. There is a uniform distribution, except at the beginning of the readings, a deviation attributed to the elimination of the 5′ adapters. This is considered normal in the method and does not alter the results. The G + C content remains constant and close to the previously detected value of 50%. D. Distribution of sequence lengths. Most of the readings were around 50 ba [file 12864_2019_5430_MOESM1_ESM.pdf]

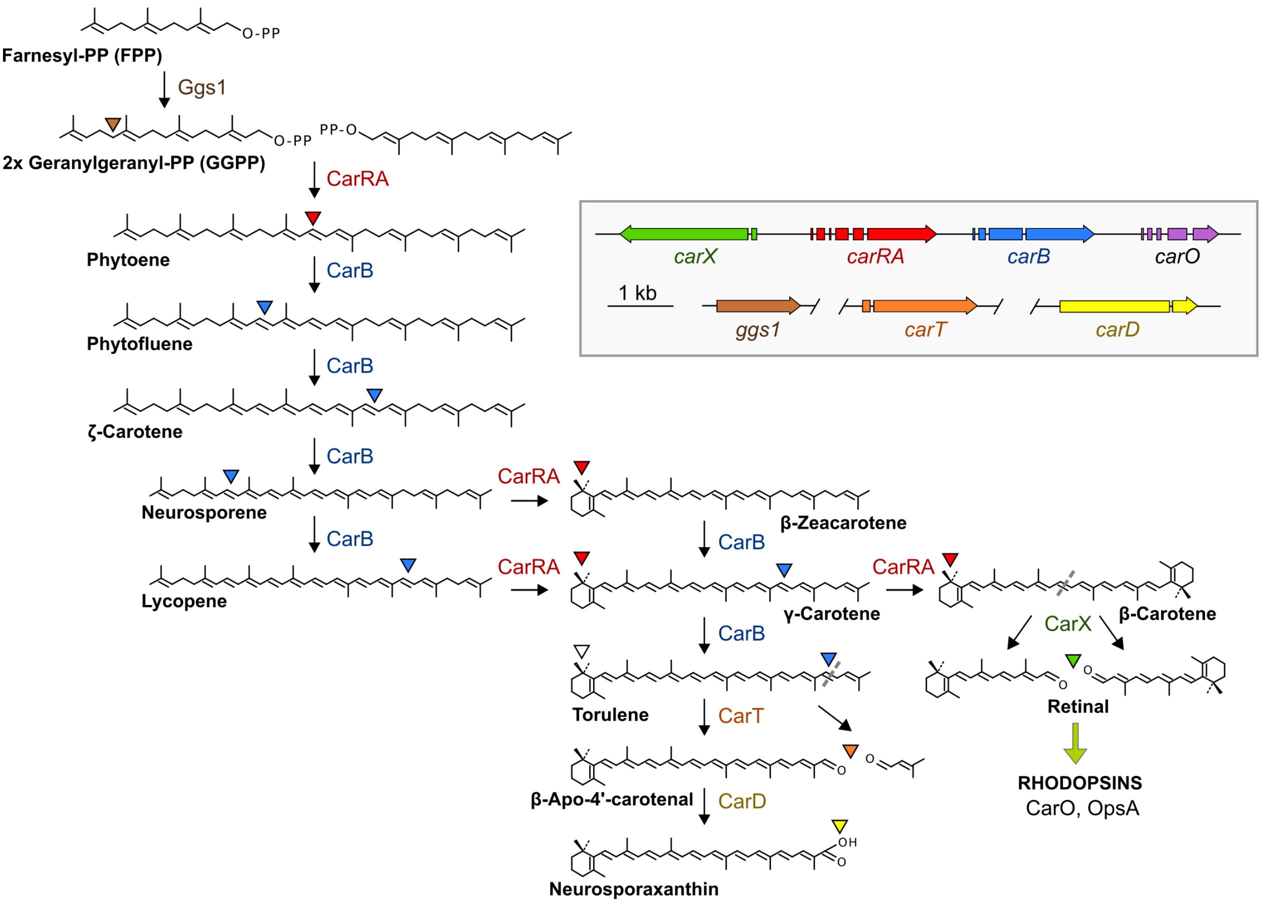

Figure S1. Biochemical reactions and enzymes involved in the metabolism of carotenoids in *Fusarium*. The arrowheads indicate the reaction sites in the molecules. The genomic organization of the structural genes is shown in the box.

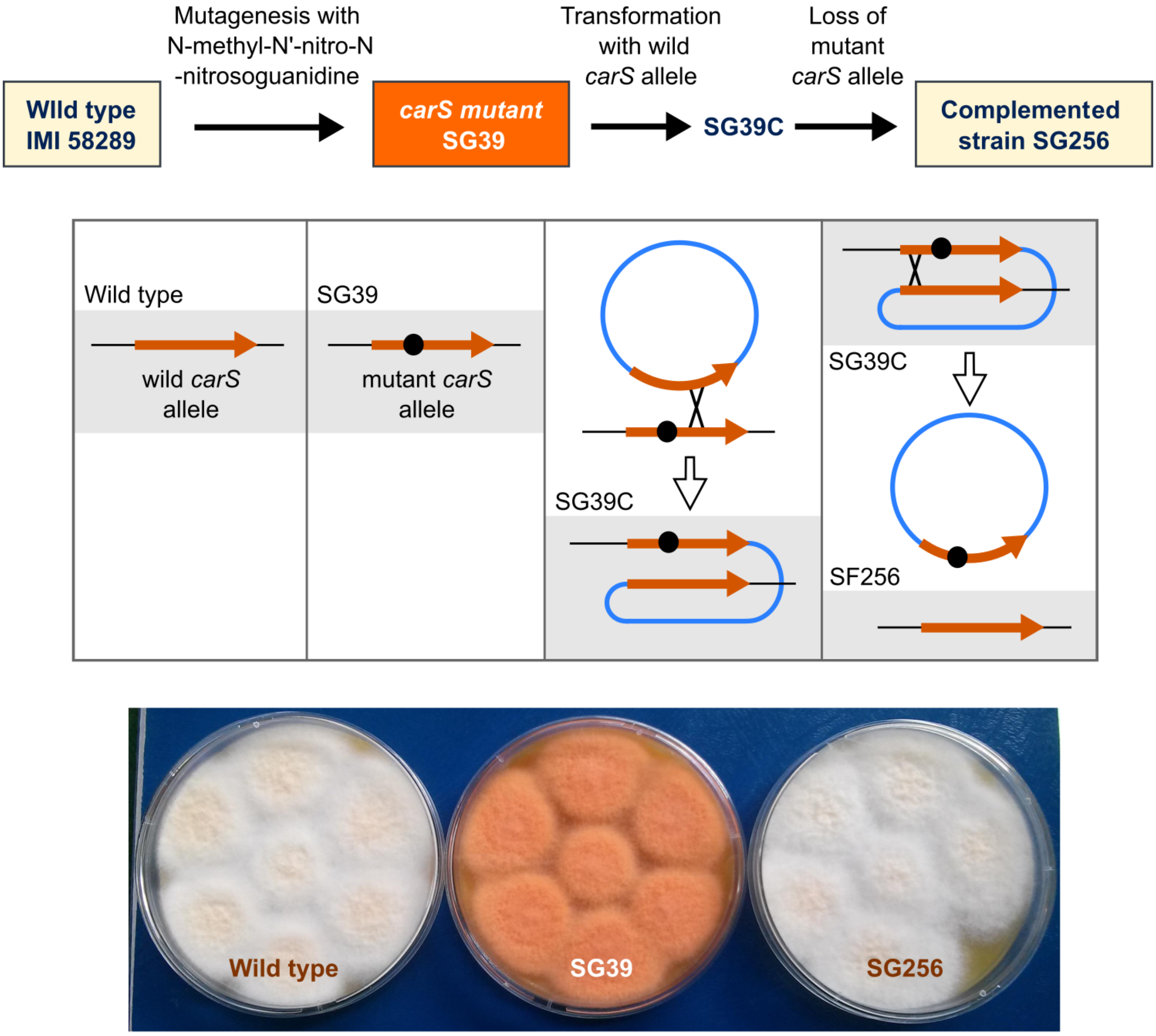

Figure S2. Molecular steps in the generation of the complemented strain SG256. The *carS* mutant SG39 was obtained by chemical mutagenesis. In a first complementation step, a plasmid containing the wild *carS* allele was introduced in the *carS locus* of SG39 by homologous recombination. This strain is unstable due to spontaneous loss of the plasmid by new recombination events. The stable complemented strain SG256 was obtained by the loss of the plasmid carrying the *carS* mutant allele.

References

Avalos J, Casadesús J, Cerdá-Olmedo E (1985) *Gibberella fujikuroi* mutants obtained with UV radiation and N-methyl-N'-nitro-N-nitrosoguanidine. Appl. Environ. Microbiol. 49:187–191.

Rodríguez-Ortiz, R., Limón, M.C., and Avalos, J. (2013). Functional analysis of the *carS* gene of *Fusarium fujikuroi*. Mol. Genet. Genomics 288, 157–173.

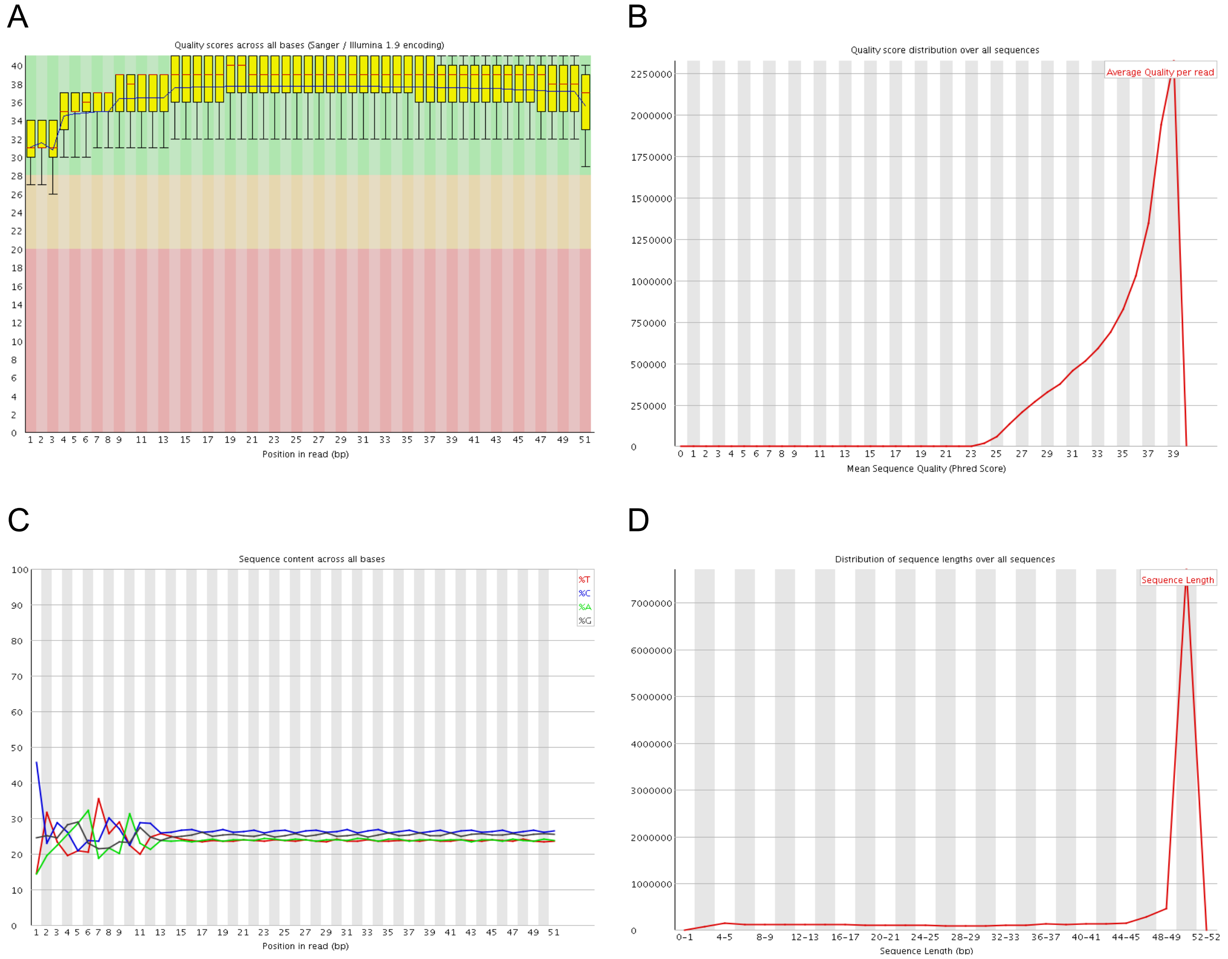

Figure S3. Quality verification according to different parameters shown through the FastQC program for a representative sample (wild type dark 1).

A. Quality representation according to the position of the nucleotide for all the readings detected in the sequencing. Our analysis corresponds to fragments about 50 bases in length, in which no appreciable quality problems were detected throughout the readings. The blue line represents the mean values and the red line the median. Practically all the readings are in the green zone, which confirms its quality.

B. Measurement of the average quality of each reading. The quality is represented in abscissa and the number of readings in ordinates. It can be seen that the average quality is higher than 30. Sequences with a more irregular distribution and values below 20 would indicate quality problems.

C. Distribution of the base content throughout the readings. There is a uniform distribution, except at the beginning of the readings, a deviation attributed to the elimination of the 5' adapters. This is considered normal in the method and does not alter the results. The G+C content remains constant and close to the previously detected value of 50%.

D. Distribution of sequence lengths. Most of the readings were around 50 bases.

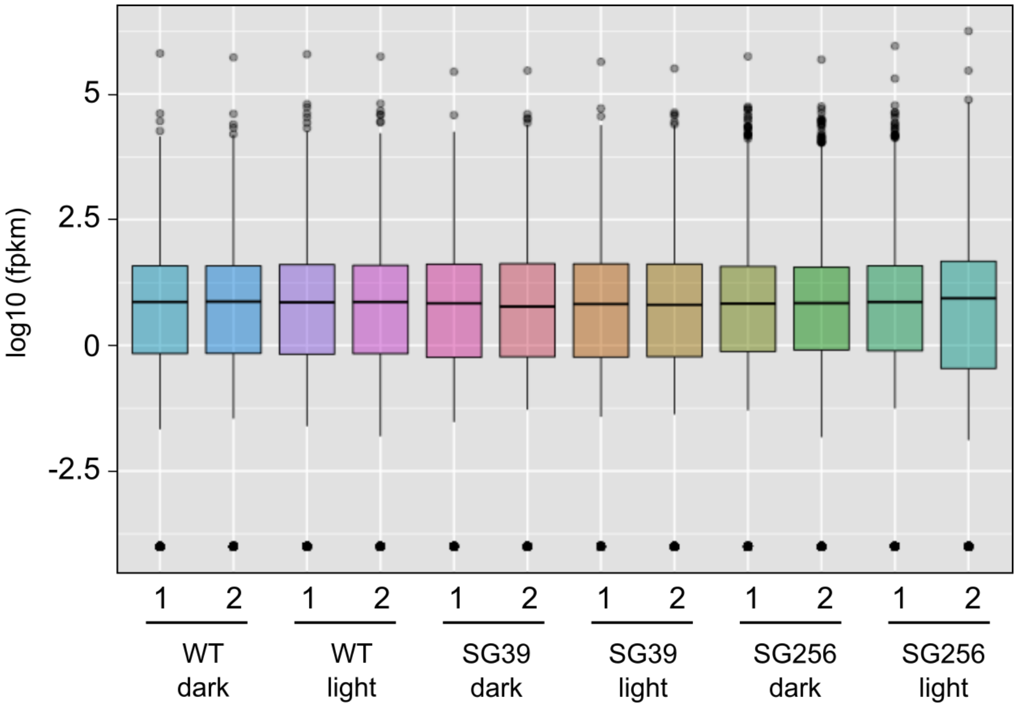

Figure S4. Boxplot representation of the samples of *F. fujikuroi* used in the study. The boxes represent 50% of the variation (±1 quartile). The lines in the boxes indicate the mean. Points indicate the farthest genes from the mean, either for activation (positive values for the Y axis) or for repression (negative values for the Y axis).

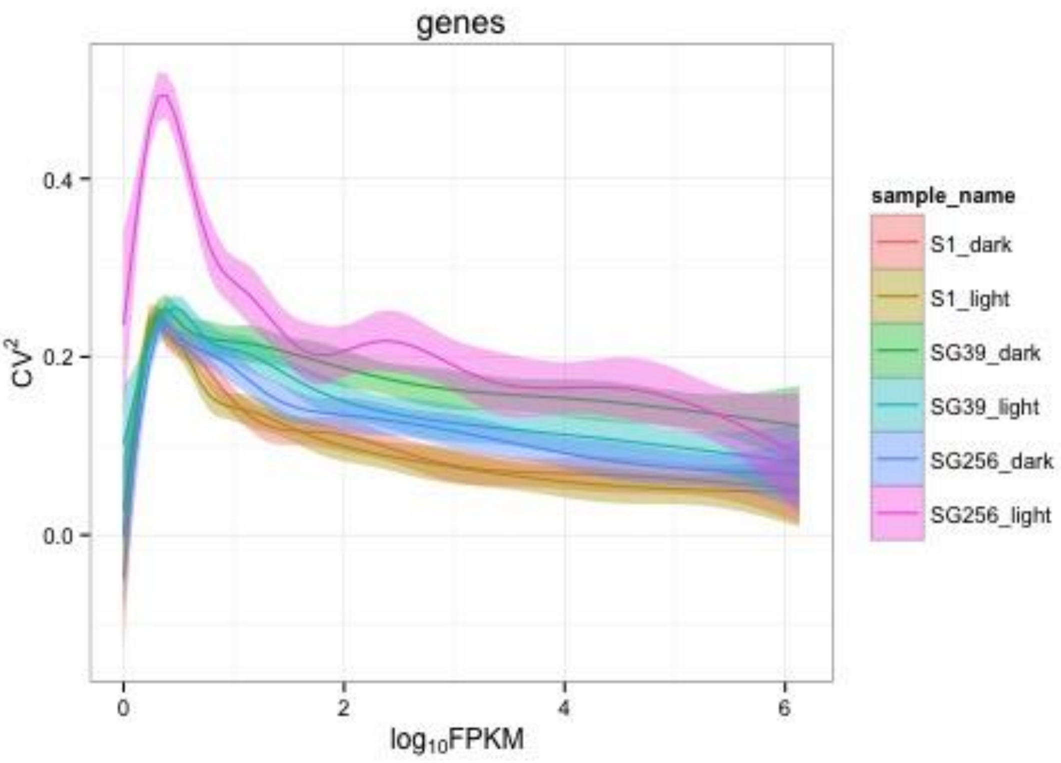

Figure S5. Global distribution of the repetition of gene expression between biological replicates in function of the values of gene expression. The internal lines represent the average variation. CV: Variation coefficient, used as a measure of the dispersion of expression values.

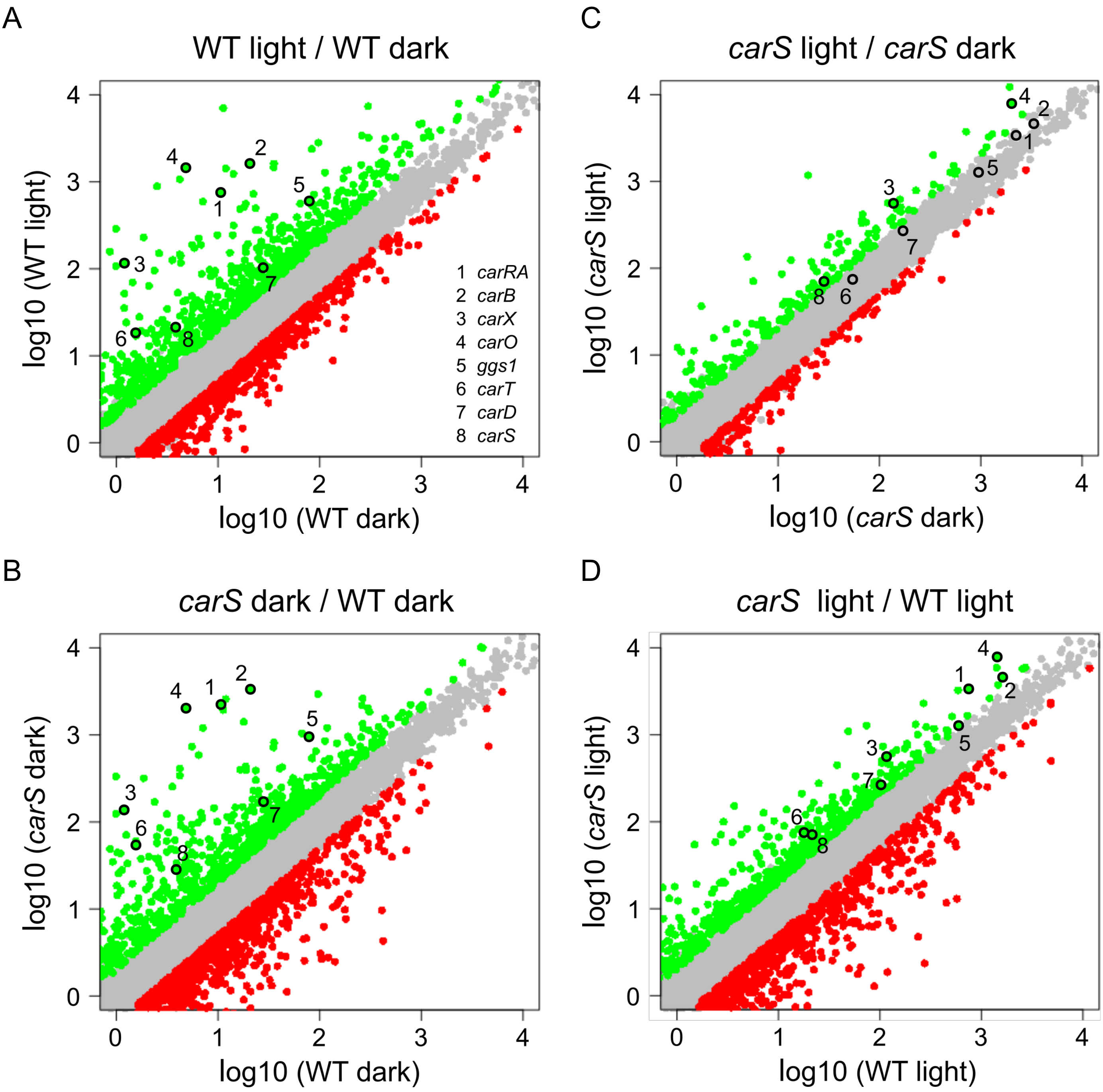

Figure S6. Scatter plot representations of the effect of light and SG39 genotype (*carS*) on transcript levels of the *F. fujikuroi* genes. The numbers indicate genes related to carotenogenesis according to the legend shown in panel WT light / WT dark.

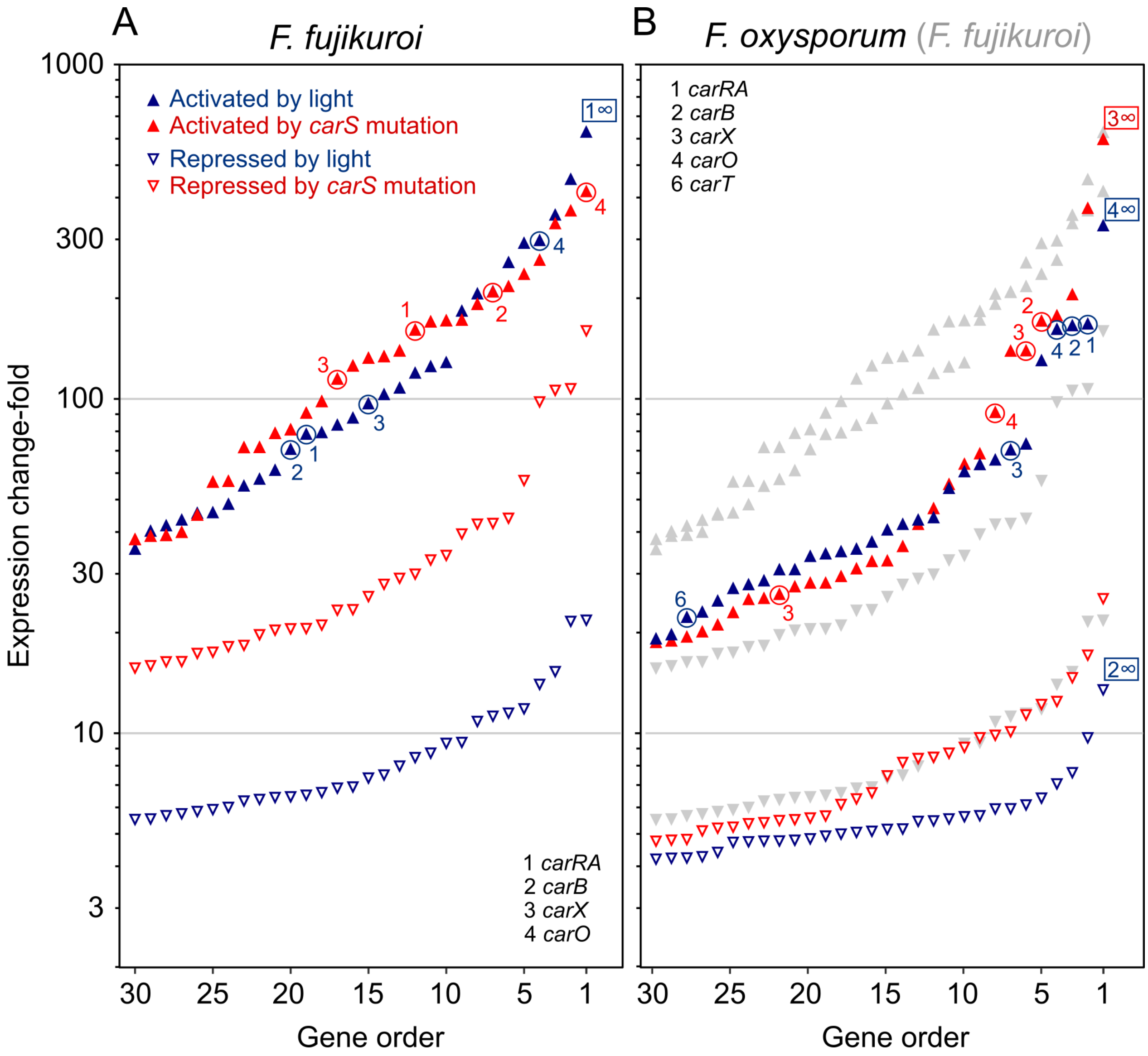

Figure S7. Graphic representation of the expression changes for the 30 genes with the higher activation (ascending arrowheads) and repression (descending arrowheads) as a result of light (blue symbols) or *carS* mutation (red symbols) in *F. fujikuroi* (A) and *F. oxysporum* (B). Genes were ordered according to increasing expression fold-changes. The genes of *F. fujikuroi* are shown in grey in panel B for a better comparison. The sets of 30 genes exclude one gene from *F. fujikuroi* and nine genes from *F. oxysporum* without detectable expression in the wild type in the dark, resulting in infinite fold change (indicated as [#∞] in a box in the top of their corresponding groups). The genes of the *car* cluster are indicated with circles and numbers according to the inner legends. In the case of induction by light in *F. oxysporum*, *carT* was also found in the represented genes.

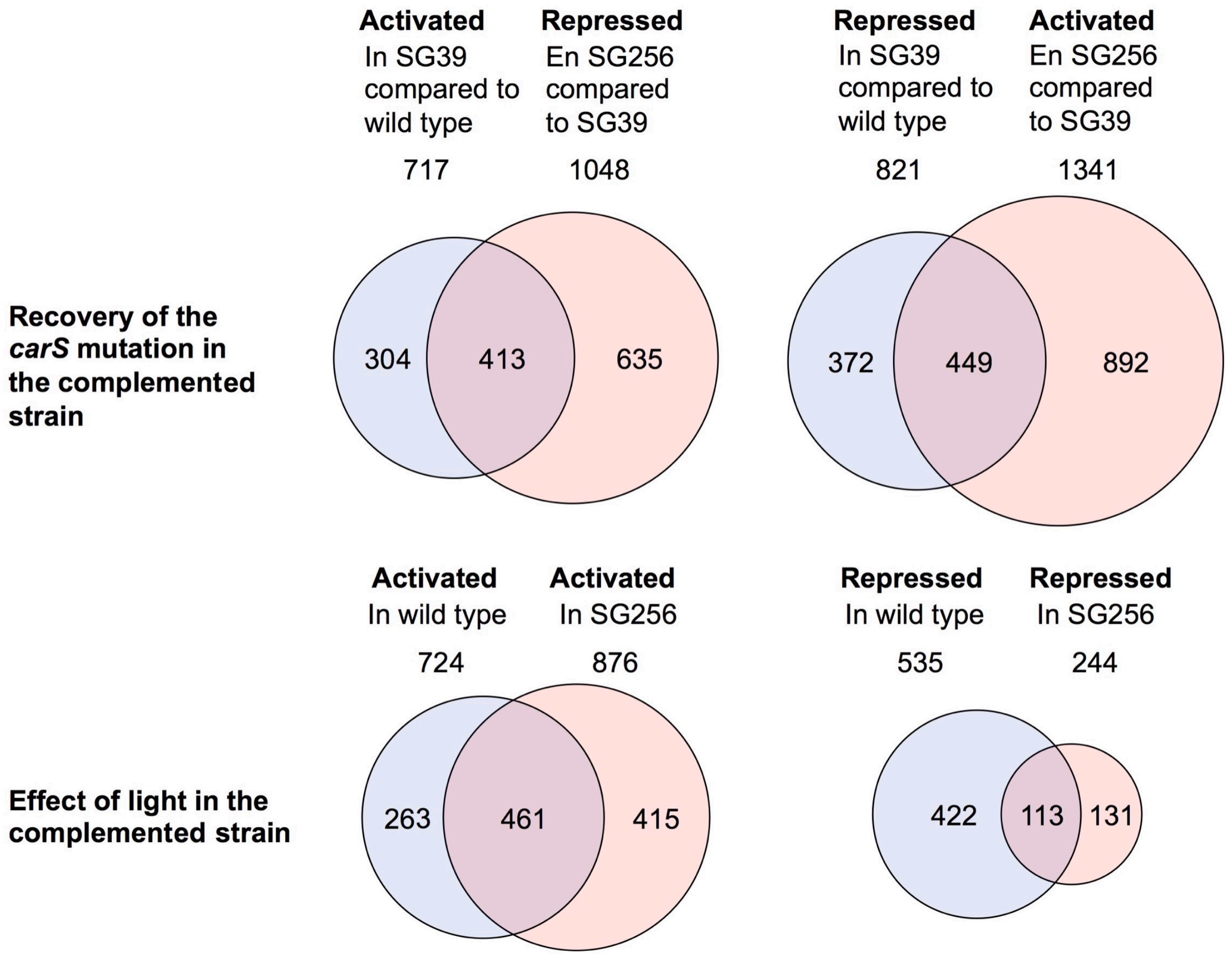

Figure S8. Recovery of wild expression pattern in the complemented strain SG256. Above: Venn diagrams representing the overlap between genes activated or repressed in SG256 in relation to the *carS* mutant in the dark. Below: Venn diagrams of matches between the genes activated and repressed by light in the wild strain and in SG256. The number of coincident genes is shown in the overlapping areas. The surfaces of the circles and the intersections are proportional to the number of genes.

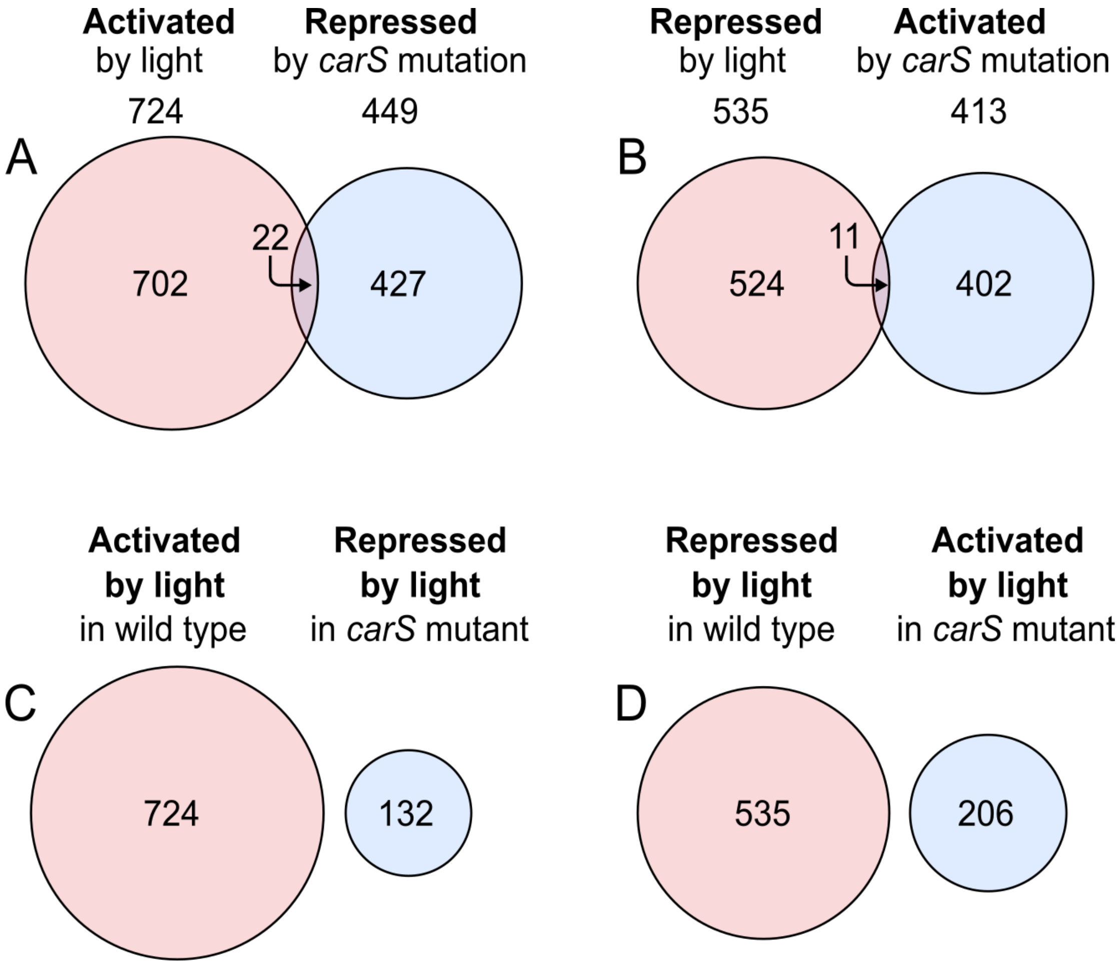

Figure S9. Venn diagrams of *F. fujikuroi* genes with opposite effects by light or by the *carS* mutation. A-B Venn diagrams of *F. fujikuroi* genes activated or repressed by light or by the *carS* mutation. The effect of the *carS* mutation was corrected with the data of the SG256 complemented strain. **C-D** Venn diagrams of *F. fujikuroi* genes activated or repressed by light in the wild type or in the *carS* mutant SG39. In all the diagrams, the numbers indicate genes that correspond to the conditions mentioned above. The surfaces of the circles are proportional to the numbers of genes. The intersections between the circles in diagrams A and B correspond to genes coinciding in the conditions compared. The set of genes repressed/activated by the *carS* mutation in dark conditions did not significantly overlap with the sets of genes activated/repressed in the wild type (WT) according to *p*-values of 0.49 (22 genes in diagram A) and 0.86 (11 genes in diagram B), respectively. There was no overlap in the sets of genes shown in diagrams C and D.

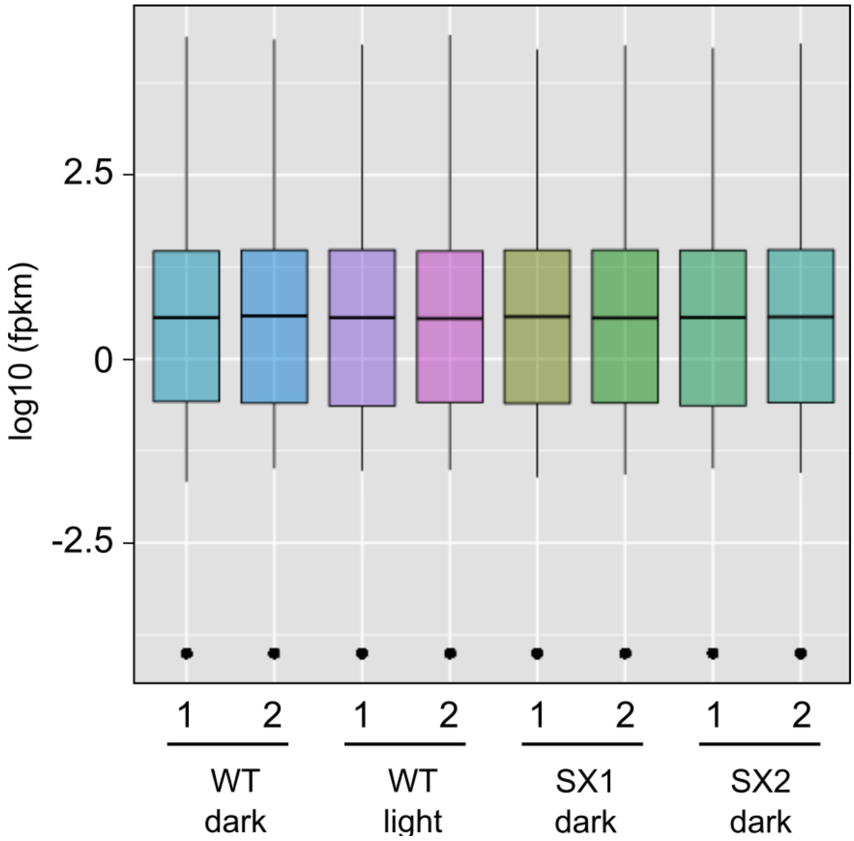

Figure S10. Boxplot representation of the samples of *F. oxysporum* used in the study. The boxes represent 50% of the variation (±1 quartile). The lines in the boxes indicate the mean. Points indicate the farthest genes from the mean, either for activation (positive values for the Y axis) or for repression (negative values for the Y axis).

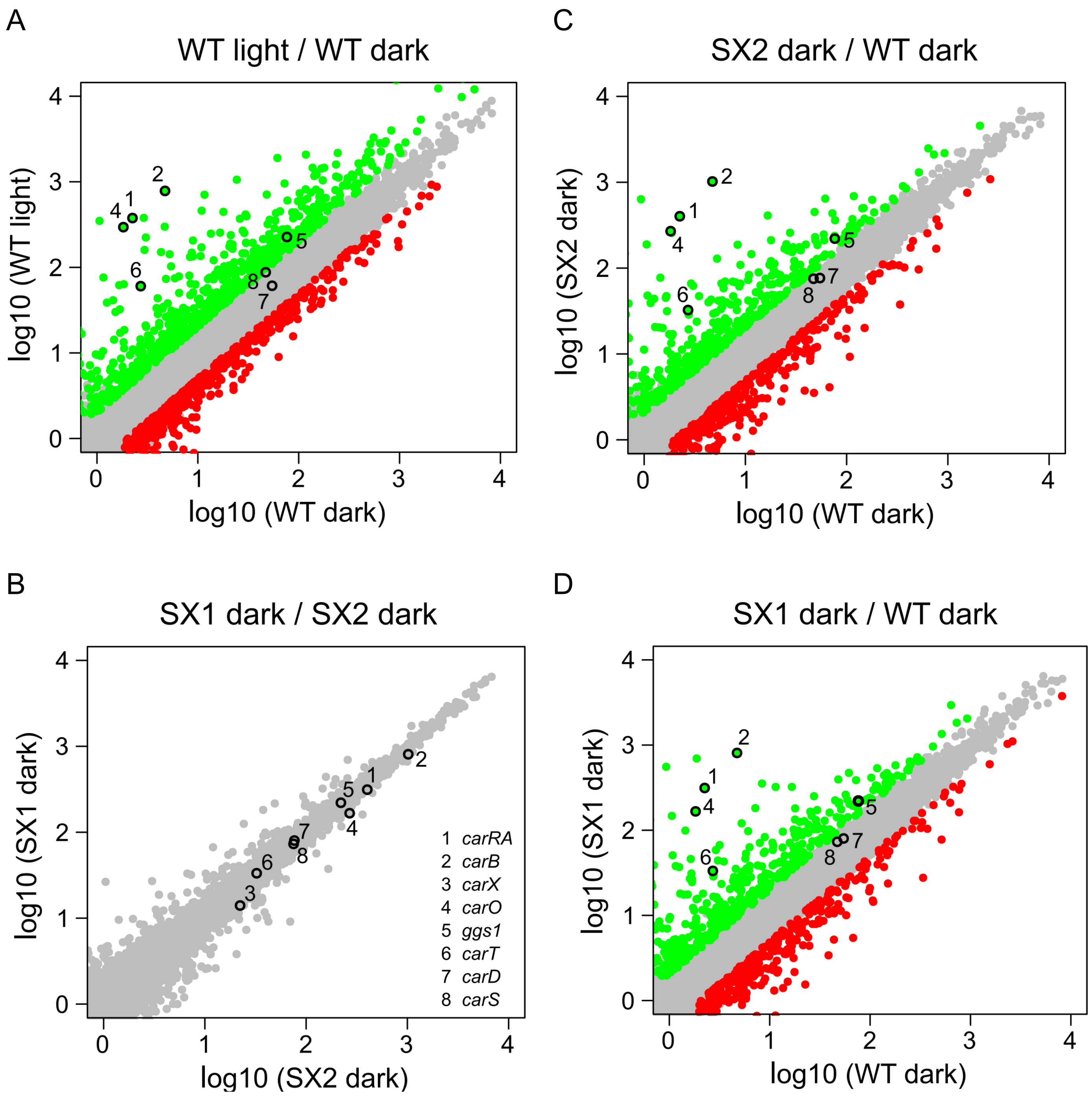

Figure S11. Scatter plot representations of the effect of light and *carS* mutation on the transcript levels of the *F. oxysporum* genes. The numbers indicate genes related to carotenogenesis according to the legend shown in panel SX1 dark / SX2 dark.
