# Supplementary material for: Comparative transcriptomic analysis unveils interactions between the regulatory CarS protein and light response in Fusarium
Source: BMC Genomics. 2019 Jan 21;20:67. doi: 10.1186/s12864-019-5430-x (PMC6340186; doi:10.1186/s12864-019-5430-x)
Supplement: Supplementary file 2 — Table S1. Basic features of the sequences used in the RNA-seq analysis described in this work. (PDF 26 kb) [file 12864_2019_5430_MOESM2_ESM.pdf]

Table S1. Basic features of the sequences used in the RNA-seq analysis described in this work

A. *F. fujikuroi*

Sample	nº readings (x 10^6)	Mapped reads (%)	G+C content (%)	Average reading length	Quality*
WT dark (1)	11.13	92.7	52	44.22	35,36
WT dark (2)	8.60	92.6	52	44.19	35.37
WT light (1)	10.35	92.8	52	44.16	35.36
WT light (2)	16.34	92.6	52	44.10	35.36
SG39 dark (1)	9,27	91.8	52	44.22	35.38
SG39 dark (2)	9.83	92.1	52	43.85	35.25
SG39 light (1)	9.55	92.3	52	44.10	35.34
SG39 light (2)	10,08	92.1	51	44,06	35,35
SG256 dark (1)	8,44	92.9	51	44.25	35.40
SG256 dark (2)	10,07	92.7	51	44.03	35.30
SG256 light (1)	8.61	92.9	51	44.21	35.39
SG256 light (2)	21.19	98.0	52	49,56	36,61

B. *F. oxysporum*

Sample	nº readings (x 10^6)	Mapped reads (%)	G+C content (%)	Average reading length	Quality
WT dark (1)	11.97	90.4	53	43.70	35.22
WT dark (2)	9.71	91.5	52	43.96	35.27
WT light (1)	8,91	91.1	52	43.76	35.19
WT light (2)	10.41	91.5	52	43.99	35.29
SX1 dark (1)	11.50	91.0	52	43.93	35.29
SX1 dark (2)	10.49	91.2	52	43.97	35.30
SX2 dark (1)	8.76	91.4	52	43.94	35.27
SX2 dark (2)	9.72	90.7	52	43.84	35.27
