# Supplementary material for: Comparative transcriptomic analysis unveils interactions between the regulatory CarS protein and light response in Fusarium
Source: BMC Genomics. 2019 Jan 21;20:67. doi: 10.1186/s12864-019-5430-x (PMC6340186; doi:10.1186/s12864-019-5430-x)
Supplement: Supplementary file 4 — Table S3. GO term enrichment in genes activated by light or by the carS mutation in F. fujikuroi (A) and F. oxysporum (B). (PDF 128 kb) [file 12864_2019_5430_MOESM4_ESM.pdf]

Table S3. GO term enrichment in genes activated by light or by the *carS* mutation in *F. fujikuroi* (Table S3A) and *F. oxysporum* (Table S3B).

Table S3A. *Fusarium fujikuroi*

**GO term enrichment in genes activated by light in the wild type**				
**GO term and description**	**Gene**	**Putative function**	**Assigned function in annotated genome**	**Fold-change**
GO:0000160 (p-value = 2.4 x 10^{-4}) Phosphorelay Signal Transduction System	FFUJ_04526 [L]		related to large-conductance mechanosensitive channel	2,01
	FFUJ_02424 [L]	mscL; large conductance mechanosensitive channel	related to nik-1 protein (Os-1p protein)	2,23
	FFUJ_02668 [L]	POLD1; DNA polymerase delta subunit 1	probable DNA-directed DNA polymerase III	2,02
	FFUJ_02280 [L]		related to histidine kinase	2,79
	FFUJ_13427 [L]		related to nik-1 protein (Os-1p protein)	2,46
	FFUJ_13182 [LL]		related to two-component histidine kinase chk-1	2,33
	FFUJ_09936 [L]		related to histidine kinase	3,16
	FFUJ_09572 [L]	RICTOR; rapamycin-insensitive companion of mTOR	related to sexual differentiation and meiosis protein ste20	3,4
	FFUJ_06191 [LS]		UP	4,47
	FFUJ_10367 [L]		related to sensory transduction histidine kinase	33,0

GO:0008152 (p-value = 0.032) Metabolic process	FFUJ_00826	DHPS, dys; deoxyhypusine synthase	probable deoxyhypusine synthase	2,24
	FFUJ_00739		UP	2,39
	FFUJ_01952		related to delta-5/delta-6 fatty acid desaturase	2,53
	FFUJ_02416	SUOX; sulfite oxidase	related to nitrate reductase	2,11
	FFUJ_02467 [S]	cynS; cyanate lyase	probable cyanate lyase	2,23
	FFUJ_02509 [S]	ASPG; 60kDa lysophospholipase	related to Lysophospholipase	2,37
	FFUJ_13182 [LL]		related to two-component histidine kinase chk-1	2,33
	FFUJ_13226	POLD4; DNA polymerase delta subunit 4	related to DNA polymerase delta subunit 4	2,22
	FFUJ_13245 [L]		probable period clock protein FRQ	2,26
	FFUJ_14513 [S]	NR; nitrate reductase (NAD(P)H)	related to nitrate reductase	5,46
	FFUJ_14666	SUOX; sulfite oxidase	related to nitrate reductase	7,09
	FFUJ_09934		related to light induced alcohol dehydrogenase Bli-4	2,07
	FFUJ_09758		UP	3,06
	FFUJ_07352	GGPS1; geranylgeranyl diphosphate synthase, type III	probable farnesyltranstransferase (al-3)	7,56
	FFUJ_06721 [L]		UP	2,34
	FFUJ_05474		UP	2,87
	FFUJ_06562	moeA; molybdopterin molybdotransferase	related to molybdopterin biosynthesis protein moeA	5,93
	FFUJ_06571 [SS]		related to 5-methylcytosine G/T mismatch-specific DNA glycosylase	5,70
	FFUJ_06163 [SS]		UP	27,68
	FFUJ_08932		probable sulfonate biosynthesis enzyme	2,01
	FFUJ_08537	RRM2; ribonucleoside-diphosphate	probable small subunit of ribonucleotide reductase	2,25

		reductase subunit M2		
	FFUJ_14913 [L]		UP	4,24
	FFUJ_12300	PLA2G4, CPLA2; cytosolic phospholipase A2	related to phospholipase A2, cytosolic	3,96
	FFUJ_10549		related to adenine phosphoribosyltransferase	3,04
	FFUJ_11222		UP	11,45
	FFUJ_11472 [S]	katE, CAT, catB, srpA; catalase	probable catalase isozyme P	54,77
GO:0065007 (p-value = 0.043) Biological Regulation	FFUJ_04526 [L]	mscL; large conductance mechanosensitive channel	related to large-conductance mechanosensitive channel	2,01
	FFUJ_04805	TMEM63; calcium permeable stress-gated cation channel	related to A.thaliana hyp1 protein	2,02
	FFUJ_04572	manA, MPI; mannose-6-phosphate isomerase	related to mannose-6-phosphate isomerase	2,77
	FFUJ_02424 [L]		related to nik-1 protein (Os-1p protein)	2,23
	FFUJ_02668 [L]	POLD1; DNA polymerase delta subunit 1	probable DNA-directed DNA polymerase III	2,02
	FFUJ_02280 [L]		related to histidine kinase	2,79
	FFUJ_13427 [L]		related to nik-1 protein (Os-1p protein)	2,46
	FFUJ_13182 [LL]		related to two-component histidine kinase chk-1	2,33
	FFUJ_13245 [L]		probable period clock protein FRQ	2,26
	FFUJ_09936 [L]		related to histidine kinase	3,16
	FFUJ_09572 [L]	RICTOR; rapamycin-insensitive companion of mTOR	related to sexual differentiation and meiosis protein ste20	3,40
	FFUJ_06982		UP	7,51
	FFUJ_06721 [L]		UP	2,34

	FFUJ_06191 [LS]		UP	4,47
	FFUJ_14913 [L]		UP	4,24
	FFUJ_10367 [L]		related to sensory transduction histidine kinase	33,02
	FFUJ_11846	trxA; thioredoxin 1	related to thioredoxin	7,14
**GO term enrichment in genes activated by *carS* mutation in darkness**				
**GO term and description**	**Gene**	**Putative function**	**Assigned function in annotated genome**	**Fold-change**
GO:0006082 (p-value = 1.1 x 10^{-3}) Organic acid metabolic process	FFUJ_02467 [L]	cynS; cyanate lyase	probable cyanate lyase	3,95
	FFUJ_02509 [L]	ASPG; 60kDa lysophospholipase	related to Lysophospholipase	2,56
	FFUJ_14513 [L]	NR; nitrate reductase (NAD(P)H)	related to nitrate reductase	4,76
	FFUJ_11472 [L]	katE, CAT, catB, srpA; catalase	probable catalase isozyme P	365,17
GO:0033554 (p-value = 0.043) Cellular Response to Stress	FFUJ_13454 [S]	HUS1; HUS1 checkpoint protein	related to mitotic and DNA damage checkpoint protein hus1	2,19
	FFUJ_06571 [SL]		related to 5-methylcytosine G/T mismatch-specific DNA glycosylase	14,39
	FFUJ_06163 [SL]		UP	18,6
GO:0050896 (p-value = 0.048) Response to stimulus	FFUJ_00436	phrB; deoxyribodipyrimidine photo-lyase	probable deoxyribodipyrimidine photo-lyase PHR	3,74
	FFUJ_13161		related to YER185w, Rta1p	31,83
	FFUJ_13454 [S]	HUS1; HUS1 checkpoint protein	related to mitotic and DNA damage checkpoint protein hus1	2,19
	FFUJ_14520		UP	5,77
	FFUJ_07428		related to phospholipid-translocating ATPase	6,72
	FFUJ_06191 [LL]		UP	5,22
	FFUJ_06571 [LS]		related to 5-methylcytosine G/T mismatch-specific DNA glycosylase	14,39

	FFUJ_06163 [LS]		UP	18,6
	FFUJ_08266	MLST8, GBL; target of rapamycin complex subunit LST8	related to phospholipid-translocating ATPase	8,73
	FFUJ_13832	INPP5B_F; inositol polyphosphate 5-phosphatase INPP5B/F	related to inositol polyphosphate 5-phosphatase ocrl-1	2,19

L an S red superindexes indicate that the gene is also found in other GO category for genes activated by light in the wild type or by the *carS* mutation, respectively

UP: uncharacterized protein

Table S3B. *Fusarium oxysporum*				
**GO term enrichment in genes activated by light in the wild type**				
**GO term and description**	**Gene**	**Putative function**	**Assigned function in annotated genome**	**Fold-change**
GO:0050896 (p-value = 4.1 x 10^{-3}) Response to stimulus	FOXG_10951		HP	18,92
	FOXG_13765 (FFUJ_09936 LL)		HP	3,55
	FOXG_05655		HP	4,99
	FOXG_06156		HP	2,43
	FOXG_06401 (FFUJ_04572 L)	manA, MPI; mannose-6-phosphate isomerase	phosphomannose isomerase type I	4,03
	FOXG_07672 (FFUJ_13161 S)		HP	28,57
	FOXG_03925 (NFf)		HP	2,32
	FOXG_07695 [L] (FFUJ_13182 LLL)		HP	2,17
	FOXG_04428		HP	2,22
	FOXG_02789 [LS] (FFUJ_06571 LSS)		HP	14,55
	FOXG_03150 [LS] (FFUJ_06163 LLS)		amidophosphoribosyltransferase	15,81
	FOXG_09065		HP	3,53
GO:0008152 (p-value = 0.033)	FOXG_00147		triacylglycerol lipase	2,44
	FOXG_16837 [S] (FFUJ_11472 LS)	katE, CAT, catB, srpA; catalase	sulfite oxidase	12,07

Metabolic process	FOXG_15936 (NFf)		HP	27,11
	FOXG_07695 [L] (FFUJ_13182 LLL)		HP	2,17
	FOXG_07759 (FFUJ_13245 LL)		HP	2,32
	FOXG_03770 [S] (FFUJ_14513 LS)	NR; nitrate reductase (NAD(P)H)	nitrate reductase (NADH)	4,09
	FOXG_04155 (FFUJ_12300 L)	PLA2G4, CPLA2; cytosolic phospholipase A2	HP	4,08
	FOXG_04186 (FFUJ_14913 LL)		HP	4,95
	FOXG_05311 (FFUJ_07352 L)	GGPS1; geranylgeranyl diphosphate synthase, type III	geranylgeranyl pyrophosphate synthase	2,96
	FOXG_10437	ndh; NADH dehydrogenase	NADH dehydrogenase	2,76
	FOXG_05039		HP	2,16
	FOXG_02789 [LS] (FFUJ_06571 LSS)		HP	14,55
	FOXG_02794 [S] (FFUJ_06562 L)	moeA; molybdopterin molybdotransferase		6,57
	FOXG_03150 [LS] (FFUJ_06163 LLS)		amidophosphoribosyltransferase	15,81
	FOXG_09097 (FFUJ_08932 L)		cysteine sulfinate desulfinase	3,63
GO:0019725 (p-value = 0.032) cellular homeostasis	FOXG_12107 (FFUJ_11846 L)	trxA; thioredoxin 1	HP	8,17
	FOXG_07890		HP	2,17
	FOXG_09451		HP	2,01

GO term enrichment in genes activated by *carS* mutation in darkness				
**GO term and description**	**Gene**	**Putative function**	**Assigned function in annotated genome**	**Fold-change**
GO:0044281 (p-value = 0.025) Small molecule metabolic process	FOXG_16837 [L] (FFUJ_11472 LS)	katE, CAT, catB, srpA; catalase	sulfite oxidase	4,33
	FOXG_03770 [L] (FFUJ_14513 LS)	NR; nitrate reductase (NAD(P)H)	nitrate reductase (NADH)	2,2
	FOXG_02794 [L] (FFUJ_06562 L)	moeA; molybdopterin molybdotransferase	HP	2,28
GO:0006281 (p-value = 0.03) DNA repair	FOXG_02789 [LL] (FFUJ_06571 LSS)		HP	25,13
	FOXG_03150 [LL] (FFUJ_06163 LLS)		amidophosphoribosyltransferase	4,5
GO:0007155 (p-value = 0.04) Cell adhesion	FOXG_14092 (NFf)		HP	2,23
	FOXG_02847 (NFf)		HP	3,59

NFf: No counterpart in *F. fujikuroi*
L an S red superindexes indicate that the gene is also found in other GO category for genes activated by light in the wild type or by the *carS* mutation, respectively
HP: Hypothetical protein
