# Supplementary material for: Comparative transcriptomic analysis unveils interactions between the regulatory CarS protein and light response in Fusarium
Source: BMC Genomics. 2019 Jan 21;20:67. doi: 10.1186/s12864-019-5430-x (PMC6340186; doi:10.1186/s12864-019-5430-x)
Supplement: Supplementary file 8 — Tables S7 and S8, Figures S12 and S13. Methodological information of the RT-qPCR procedure. (PDF 672 kb) [file 12864_2019_5430_MOESM8_ESM.pdf]

Additional File 8. Methodological information of the RT-qPCR procedure

Table S7. Nucleic acid extraction, reverse transcription and RT-qPCR methodological details.

Nucleic acid extraction

Procedure and/or instrumentation	For RNAseq samples (RT-qPCR data in Fig. 4): Trizol extraction with Trizol reagent (Invitrogen) of mycelia ground in a mortar with liquid nitrogen.<br>For light exposure experiments (RT-qPCR data in Fig. 1): Extraction with RNeasy RNA isolation kit (Qiagen, Chatsworth, CA, USA) of mycelia ground in two 30'' pulses in a FastPrep-24 device (MP Biomedicals, Irvine, CA).
DNase treatment	Achieved with NucleoSpin RNA kit, using manufacturer instructions. 2.5 µg of RNA were treated with 1 µl DNAse (USB). Incubation: 15' at 25º. The reaction was finished by addition of 1µl STOP solution (provided by the kit)
Contamination and RNA integrity assessment	Visual inspection on gel electrophoresis
Nucleic acid quantification and yield	With a Nanodrop equipment (Coleman Technologies Inc.).<br>Concentrations of RNA samples indicated in Table S8.<br>Total yields in the range 20-80 µg

Reverse transcription

Complete reaction conditions	10 µl RNA<br>1 µl dT primer (50 µM)<br>2 µl water<br>4 µl buffer<br>2 µl dNTPs 10 mM each<br>0.5 µl Protector RNase Inhibitor (40 U/µl)<br>0.5 µl Reverse transcriptase (20 U/µl)
Amount of RNA and reaction volume	1,5 µg RNA in 20 µl
Priming oligonucleotide (if using GSP) and concentration	Anchored Oligo (dT)18 Primer. 50 µM (1 µl in 20 µl of reaction)
Reverse transcriptase and concentration	Transcriptor Reverse Transcriptase. 0,5 µl (20 U/µl) in 20 µl of reaction
Temperature and time	Primer binding: 10' at 60º. Reaction: 10' at 25º + 30' at 55º

Manufacturer of reagents and catalogue numbers	Transcriptor First Strand cDNA Synthesis Kit, Roche 04897030001

qPCR protocol

Complete reaction conditions	2 µl cDNA<br>2.6 µl water<br>0.4 µl primers mix (10 µM)<br>5 µl LightCycler® 480 SYBR Green I Mastermix (Roche) (ready to use 2x mix)
Reaction volume and amount of cDNA/DNA	10 µl of reaction<br>2 µl containing *ca.* 50 ng cDNA
Primer, (probe), Mg2+, and dNTP concentrations	Primers: 0.4 µl (10 µM). Primers were synthesized by StabVIDA (Oeiras, Portugal). Purification: standard desalting. Mg^{2+} and dNTP concentrations as provided in LightCycler® 480 SYBR Green I Mastermix.
Polymerase identity and concentration	LightCycler® 480 SYBR Green I Master. Buffer provided by the kit.
Additives (SYBR Green I, DMSO, and so forth)	Only those present in the LightCycler® 480 SYBR Green I Master (Roche)
Manufacturer of plates/tubes and catalog number	LightCycler 480 Multiwell Plate 384, white,<br>Ref. 04729749 001
Complete thermocycling parameters	Preincubation: 95ºC 5 min, ramp rate 4.8ºC/s<br>Amplification: 45 cycles, 10 s 95ºC with ramp rate 4.8ºC/s, 10 s 60ºC with ramp rate 2.5ºC /s, 10 s 72ºC with ramp rate 4.8ºC/s<br>Melting curve: 5 s 95ºC with ramp rate 4.8ºC/s, 60 s 65ºC with ramp rate 2.5ºC /s, increase to 97ºC with ramp rate 0.11ºC/s with 5 data acquisitions per ºC<br>Final step: cooling to 40ºC during 30 s with ramp rate 2.5ºC /s

Data analysis

qPCR analysis program (source, version)	LightCycler 480 Software release 1.5.0 SP3
Method of Cq determination	Second Derivative Maximum

Outlier identification and disposition	No outliers considered in this work
Results for NTCs	No detectable amplification in all tests
Description of normalization method	ΔΔCT -Method
Number and stage (reverse transcription or qPCR) of technical replicates	Three technical replicates of each qPCR reaction
Repeatability (intra-assay variation)	All values in the range of ∓ 1 Cq values
Statistical methods for results significance	Unpaired T- test
Software (source, version)	Graphpad Prism 7.0

Table S8. Concentration and A260/A280 absorbance ratios of RNA samples used in the RT-qPCR experiments.

Data		Sample	ng/µl	260/280
Figure 1 (Effect of light pulse duration on the transcript levels of *carRA* and *carB* genes	Biological replicate 1	Dark	846	N.D.
		5’ light	1131	N.D.
		15’ light	1461	N.D.
		30’ light	919	N.D.
		60’ light	680	N.D.
	Biological replicate 2	Dark	1138	2.02
		5’ light	1371	2.01
		15’ light	734	2.00
		30’ light	841	2.00
		60’ light	1393	1.99
	Biological replicate 3	Dark	1174	1.99
		5’ light	716	2.01
		15’ light	1084	2.02
		30’ light	1302	2.00
		60’ light	1063	2.01
Figure 4 (Effect of light and *carS* mutation on several putative stress-related genes.	Biological replicate 1	Wild type dark	1496	2.09
		SG39 dark	1370	2.10
		SG256 dark	849	2.11
		Wild type light	1308	2.10
		SG39 light	794	2.11
		SG256 light	1287	2.10
	Biological replicate 2	Wild type dark	1247	2.10
		SG39 dark	400	2.08
		SG256 dark	1295	2.10
		Wild type light	1382	2.11
		SG39 light	1071	2.11
		SG256 light	1630	2.10

N.D.: Not determined

Figure S12. qPCR validation curves and resulting efficiency values.

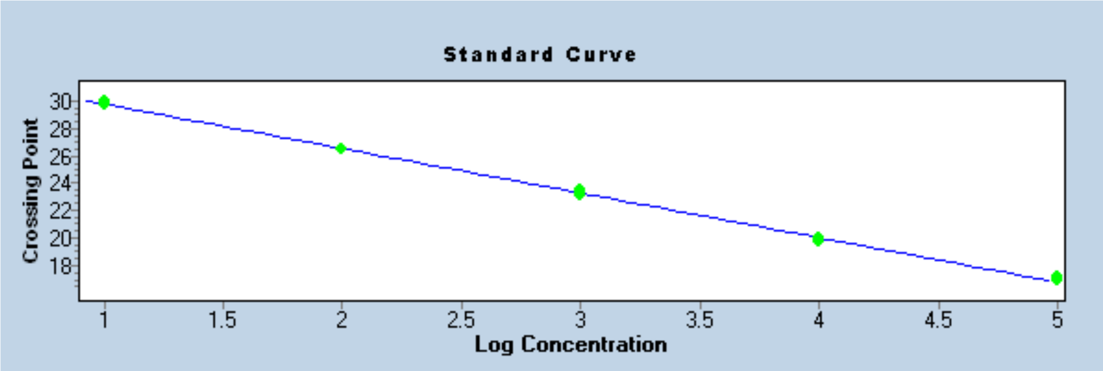

**FFUJ_13490**	R^2 = 0.9987	Error: 0.0324	Slope: -3.266	Efficiency: 2.024	Yintercept: 33.09

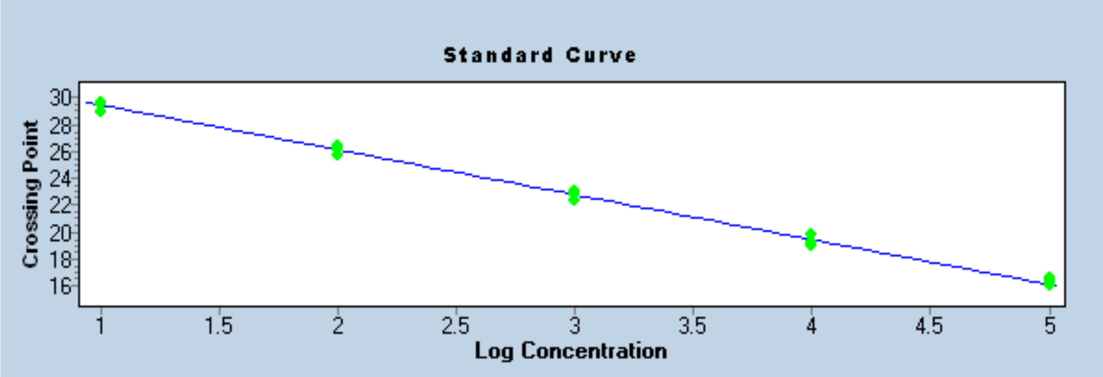

**FFUJ_11803**	R^2 = 0.996	Error: 0.0381	Slope: -3.321	Efficiency: 2.000	Yintercept: 32.76

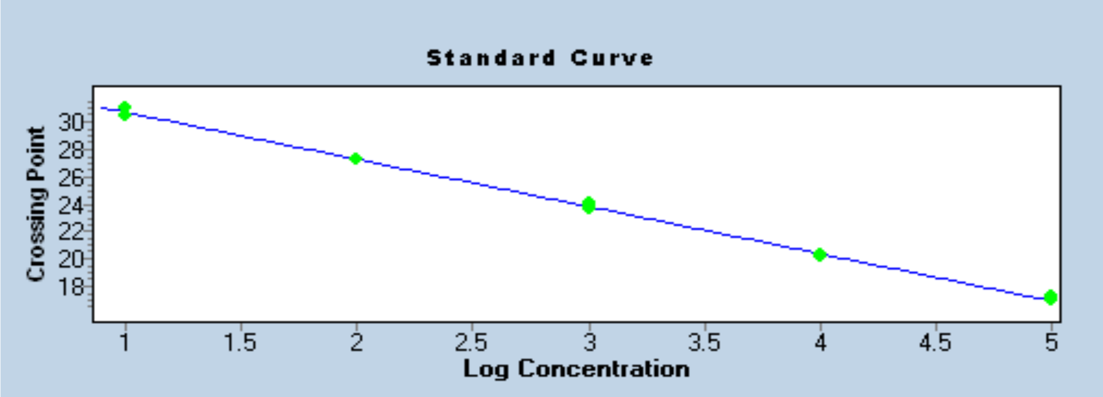

**FFUJ_11802**	R^2 = 0.9985	Error: 0.0143	Slope: -3.423	Efficiency: 1.960	Yintercept: 33.94

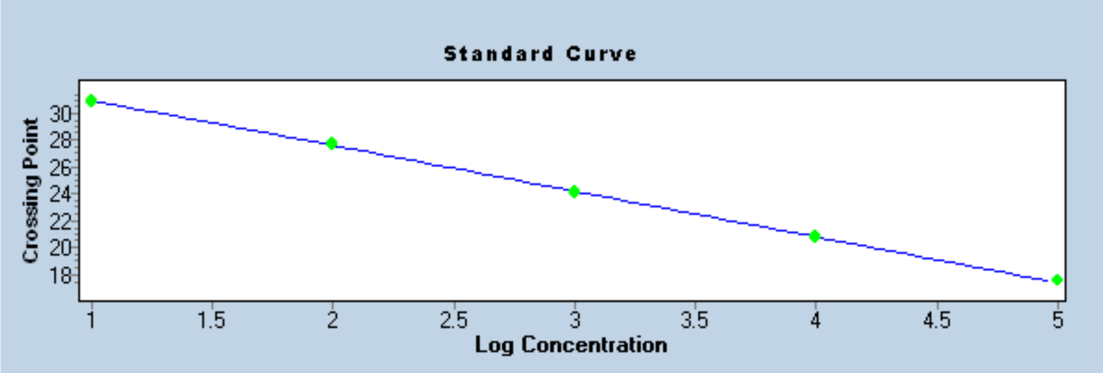

**FFUJ_10321**	R^2 = 0.9995	Error: 0.0133	Slope: -3.396	Efficiency: 1.970	Yintercept: 34.30

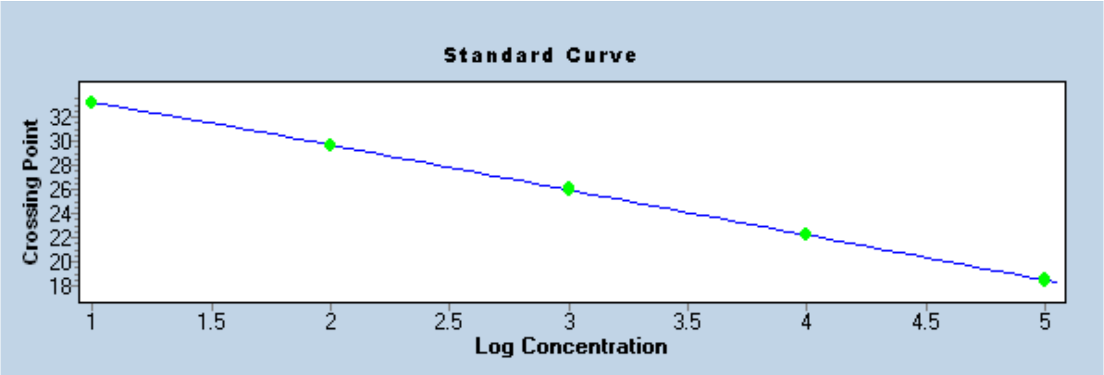

**FFUJ_09320**	R^2 = 0.9995	Error: 0.00469	Slope: -3.721	Efficiency: 1.859	Yintercept: 36.95

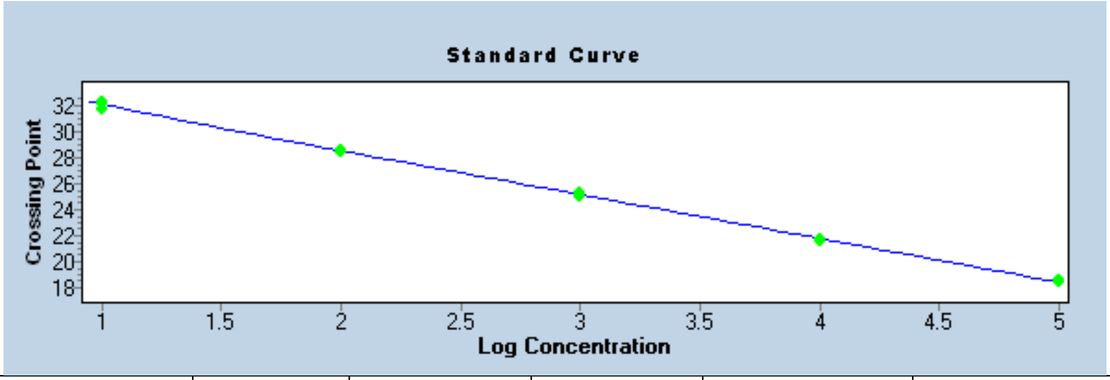

**FFUJ_09119**	R^2 = 0.9988	Error: 0.0167	Slope: -3.351	Efficiency: 1.988	Yintercept: 35.44

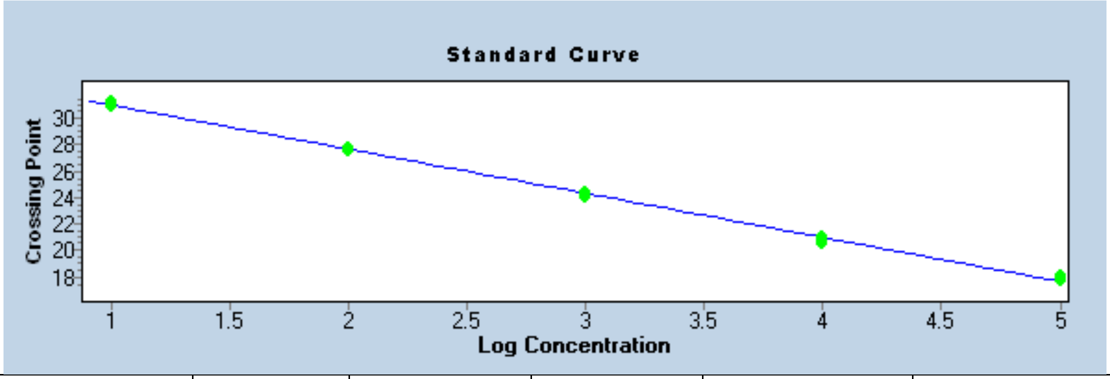

**FFUJ_05128**	R^2 = 0.9976	Error: 0.0593	Slope: -3.318	Efficiency: 2.002	Yintercept: 34.28

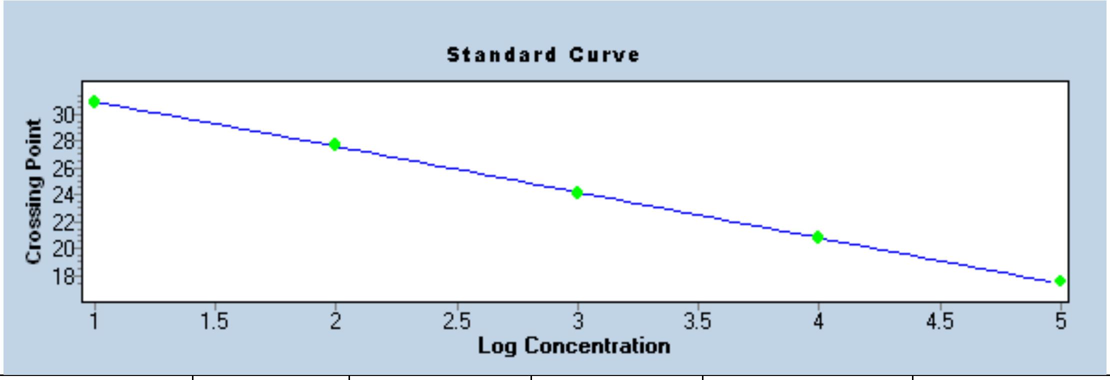

**FFUJ_04397**	R^2 = 0.9996	Error: 0.0181	Slope: -3.343	Efficiency: 1.991	Yintercept: 33.08

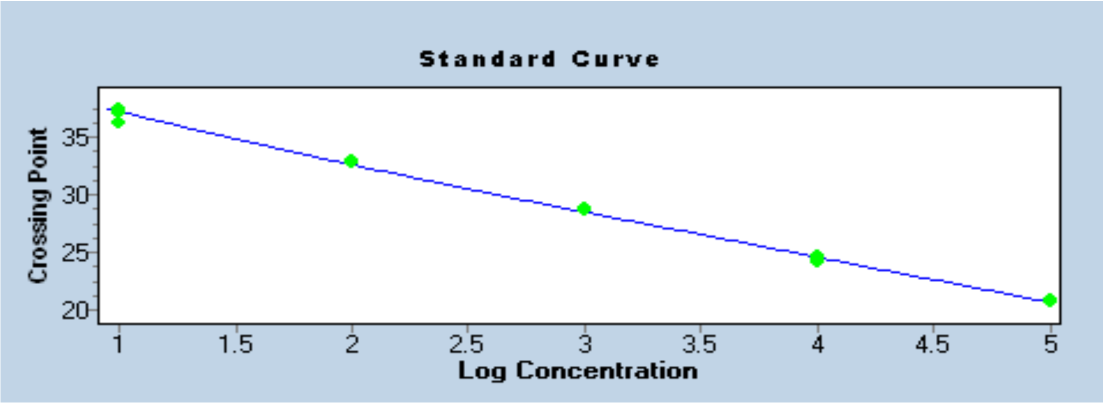

**FFUJ_03407**	R^2 = 0.9968	Error: 0.0219	Slope: -3.897	Efficiency: 1.806	Yintercept: 40.96

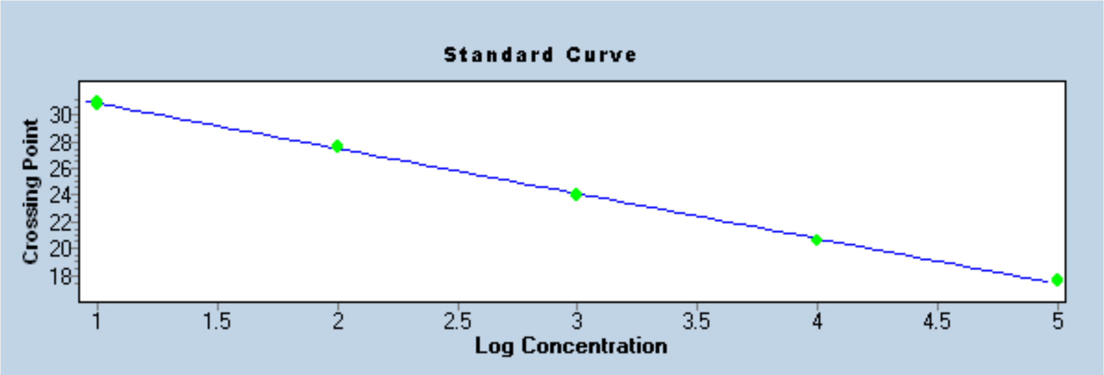

**FFUJ_1993**	R^2 = 0.9991	Error: 0.0133	Slope: -3.352	Efficiency: 1.988	Yintercept: 34.18

Figure S13. Melting curves in the samples for RT-qPCR data in Figures 1 and 4.

A. Data from Fig. 1

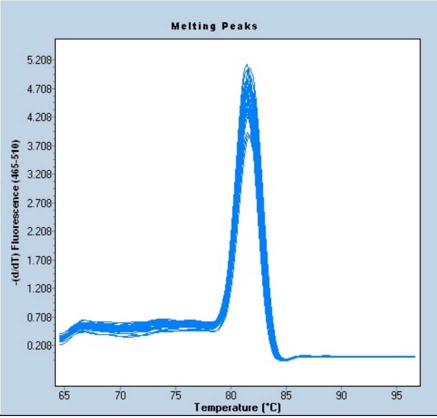

FFUJ_13490

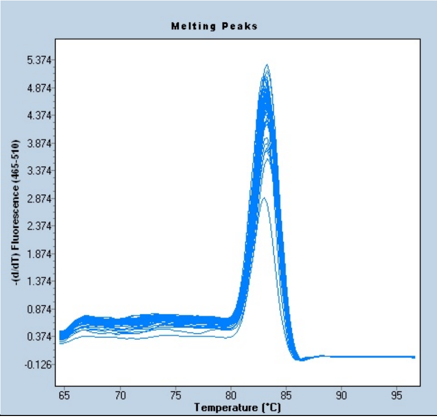

FFUJ_04397

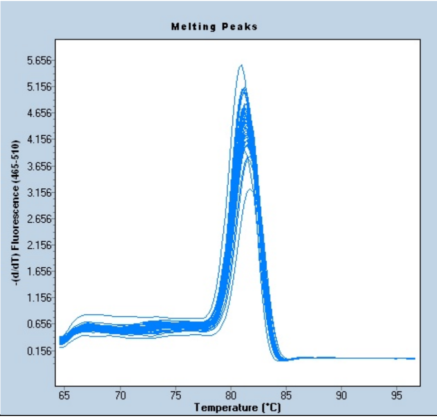

FFUJ_11802 (*carRA*)

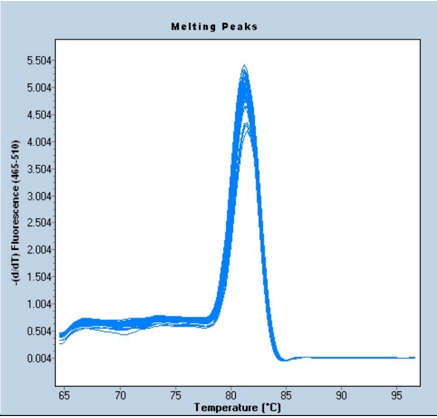

FFUJ_11803 (*carB*)

B. Data from Fig. 4

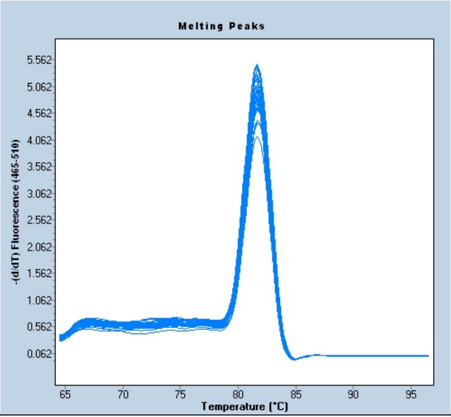

FFUJ_13490

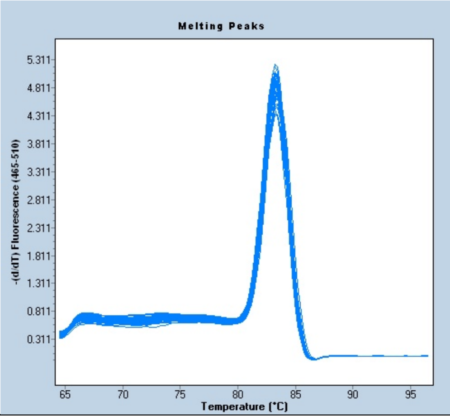

FFUJ_04397

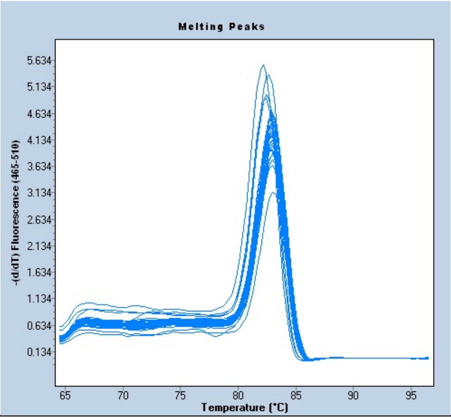

FFUJ_10321

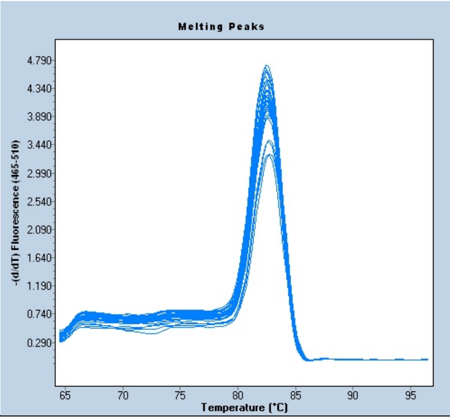

FFUJ_09320

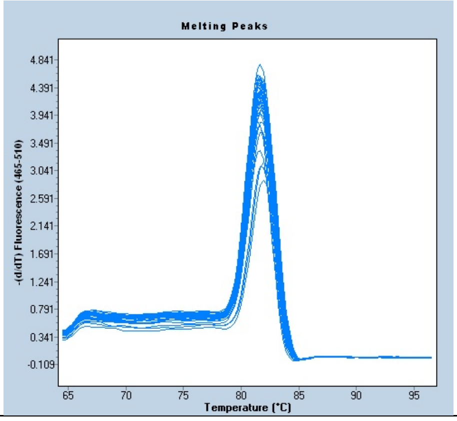

FFUJ_09119

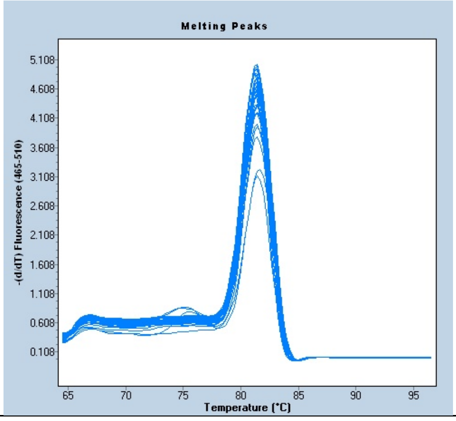

FFUJ_05128

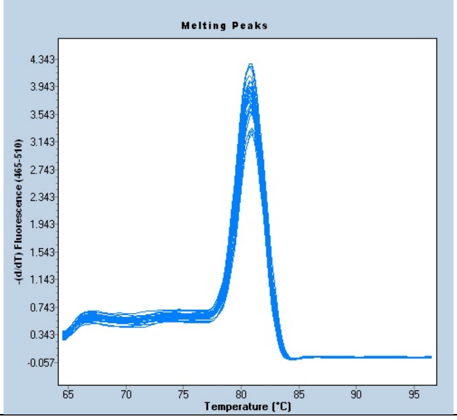

FFUJ_01993

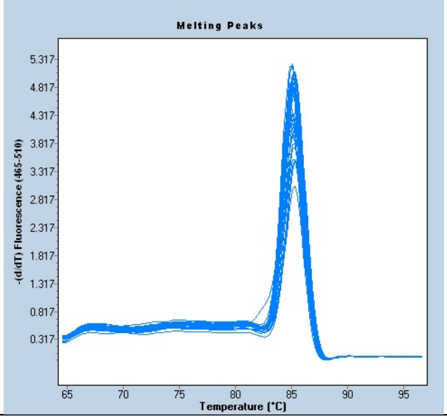

FFUJ_03407
