# Supplementary material for: Comparative transcriptomic analysis unveils interactions between the regulatory CarS protein and light response in Fusarium
Source: BMC Genomics. 2019 Jan 21;20:67. doi: 10.1186/s12864-019-5430-x (PMC6340186; doi:10.1186/s12864-019-5430-x)
Supplement: Supplementary file 9 — Sequences of the genes used in the RT-qPCR experiments and amplicon locations. (PDF 68 kb) [file 12864_2019_5430_MOESM9_ESM.pdf]

Additional file 9. Sequences of the genes used in the RT-qPCR experiments and amplicon locations. Introns are indicated in small letters in red. Amplicons are indicated in underlined letters in blue.

FFUJ_09320

ATGTCGGCCACTTCCTTCCTACGAAGTGCGATCCTCCTCGCTACCGTGGGTGTCGTCAGCGCTGTACCCGTTG
TATCAGAGGCGCAGACGACAGTCTCATCCTCTGGTCCCGCTGGCACACCTCGACCCTATAGCTTCGTGACTTC
TCACGGCCCCTTCAAGGGAACACCTACCACCACCGGAGCACTCTCAACTACAGTTCTTGCATCAGCTATTCCT
GAGCGTCCTCCGCCCCCAGGATCTTTCGACTACCCTGCCAATGGCAAGTTGAACGCTCCCCAGCCTGCACCCCT
ACACCCCAGACGGCGGTGTCGGCACCAATGGCTCTGCACCTGTATATCGCGTCCAGAGCGACTTTGACTACCA
ATCCCTCGCTCTGGCTCTGTACCAAGAGTGGATTGAACTCGATCTCTTCCGCTGGGGTCTCGCAACTTTCTCA
AAGGAAGAGTTCGACGCCTACGGCAT**CAACGATGAGGACCGCTTTCTCATCGAGCACATGGCCCGTCAAGAAA**
TCGGCCACGCGACTGTGATTAGCAACATGCTCGGCCCCGAAGCCCCGAAGCAGTGCACGTACAATTACCCCGT
GTCCAACGTCCCCGAGTTCATCGACTTCAACCAGAAGCTCACGCGCTGGGGCGAGTCAGGCGTGTACGGCTTC
CTGCCTCACTTGAACTCTGGACCTGCGGCGCAGTTACTGCTTCAGAGCATTACAATTGAGGCGAGGCAGCAGA
TGATCTTTAGGCAGTTTGGCGGTACGTTTGCTATGCCTGAGTGGCATACGCCTGGTATTCCGCAAAGCTGGGC
GTGGACTTTGTTGGCGCCATATATCTCGAGCTGCCCTTATAATCAGACGAGACTTGTGTGGCAGAACTTTCCT
TCGTTGAATATTCTAAACCAGCCCAACCCTGCGCGCATCAATGGGTCCGCGGTATGGAATGAGACGACTGGTG
GATATGCGAACACTTTGTCTACCAAGGATATCGATGACGATGAGCTCTGCGTCAACGCTACTAAGACCGGAGA
GAACTGTAAGGCCGCAATTACTCACAACCGCACCATCCCTCTCTCCTACCCCGGTCGTCAGGTATTCCTGAAC
TGGGATGCACCCGGCAAGCCTGTCGGCCCCAACAACAGCTACATCACCTCCACCAACGTCAAGGAGCCCAAGT
TCGCCGCATGGGTCTCTCAGCTCAATGTTACATACTCTCCTCTCCAGAACATCAGCCTCGAGGACAGAACGGC
TTATACCATCCAGCCTAATGTTTCAACTTGGCAGGGTGATCCGGCTATCAACGGAACTATGTTCCTCGCTTTG
ACGGATACCGACCTCTATGTCACGCCTTATAACCTTACGATGATCAATCCTCACGTTGCTGCTCTGGCTGTTT
ACCAGGCTGGTTAA

FFUJ_05128

ATG**GCGCCTTCAGCAACAGACACCTCCTTCGCAGCCCACGTAGCAAATCAATTCCAATCCTACGAATCAGACC**
GTCAAGCCACTTCCAAAGAAACACTCTACACCACCTCCAACGGCGTCCCCATCGCACACCCCTATGAGACCCA
ACGAGCCGGTGAAAATGGCCCTCTTCTCCTACAGGACTTCCACCTGATCGATCTCCTCTCACACTTTGACCGC
GAGCGTATTCCCGAGCGTGTTGTTCACGCAAAGGGAAGTGGTGCGCATGGTTACTTTGAGTGTACGGACCCGT
TAGATGATCTGTGTCTGGCGGATATCCTGTCTGAGAAGGGGAAGAAATGTTCGGTTAGTATGAGGTTTTCGAC
GGTGGGTGGTGAGTCGGGATCGCATGATATGGCGAGAGATCCTAGGGGGTTTTCGGTCAAGATGAGGACTGAT
GAGGGT**gtaagtgatctaagtgaaatgctgtggtgagatgctgatttgtgca**gAACTGGGATATGGTCTTCAA
CAACACGCCTGTTTTTTTCCTGAGAGATCCCGCCAAGTTTCCTCACTTTATCCATACTCAAAAGCGAGACCCA
A**gtaagttttcatatgatttaattgttgtttatgatcatgggctaatgagtaag**GCACTCATCTCACCCACGC
CGACGACTCATTCATGTTCTGGGACTACCTCTCCCAGAACCCCGAGTCAATCCACCAAGTCATGATCCTCATG
GGCGATAGGGGGATTCCAAAGGGGTACCGAAAGATGAACGGCTACGCTGGCCATACTTTCAAGCTTGTTAACA
AGGCTGGCGAATGGGTCTACTGTCAGATTCACCTCAAGTCCATGCAGGGCATCGACTTCGTCACCCAGGAGGA
CTCAGCCGACTACTCCCCCGATTTCTCCCAAAAGGATCTCTATGAAGCAATTCAGAACGGAGATTATCCTAAA
TGGACTCTTGAAGTTCAGACCATGACCCCCAAGGAAGCCGAGGAACTGTGGGAGAAGCAAAAGATCAACGTCT
TTGACCTGACGCACGTCTGGCCTCAGAAGCAATTCCCACTCCGAAAGGTTGGAGAGTTTACTCTGAATGAGAA
CGCAATCAACTACTTTGCCGAAGTTGAGCAGATCGCTTTCAATCCTTCTCATCTTCCTCCTGGAGTCGAGCCA
TCGGCTGATCCCGTTCTCCAATCCCGGCTGTTCTCTTACCCAGACACCCATCGCCATCGTCTCGGTGTAAACT
ACCAGCAACTTCCCGTCAACGCGACTAGAACAGGTTATCAATTCGGCAACTTCCAGCGCGACGGCCAAATGGC
ATTCTATAACCAGGGCGCACGACCGAACTATCTCTCCTCCATTGATCCTATCAAGTTCAAGTCCCGCGCTGTG
GATCTAGACAAGACCCATGGCCATTTCACTGGGGAAGCTATTACGTTCCTCACCGAAATCCGACCAGAGGACT
TTAACGCCCCTCGCGCACTTTGGAGAAATGTATTTGATGAGCCAGCTCGTGAGCGTTTCATCAACAATGTCAC
AGGCAAGATGAAGCTGTGCAAGCAGGAAGAGCCTTTGAAGCGACAGATTGCTATCTTCCGAGAGGTTGACCCT
GAGATTGCGGAGAGATTGGAGAAGTCAACTGGCATCAAGGGATACGATGGAATTGCCAATATGTCCTTCAACG
GTACACATAACGGTATGGCTTCTGGAAAGAGTAGCAGTGCCAATGGAATTATCGACAAGAACAAGACTCCTGT
TGCACGCCAAGGTGCGCCTGGTTCTGGGGCTCATGGCAGTATGGTAACAAACGGAAAGTCAAACGGTGTAAAC
GGACATTAA

FFUJ_009119

ATGTC**CCCGGAACCGTTCAATACTGATCTTGAGCACGACGGCGCAGGACTGATGGCTCAGAACGGAGATAA**AG
GTAGAGATGGGCGGAAGAAGGTGTTGGTTGTAGGTGCTGGTGCAGCTGG**gtaagttgatttcaacccataatt**
ctggatgatactgacaaaaacagCATGTCTACTGCTTACCATCTTTCGGAACATTCAGACAAATTCGACGTGA
CCCTGATTGATGCAGTTGATTACTGCGGCGGTCAAGCATTCTCCATTCCGATTGACAAGGAACGCCATGGAGC
TTCCTGGTGCAACCAAGGTGTCCAAGGAGGATCTTACATCTTTCATCATACCGTCACTATGTTCTCCCGTCAG
GGCTATCACGCAGACCCTTGCAACCTGCACGTTTCATTCGGCAAAGACGAGACGTTCTGGAATAACGTATTTC
CAACTCAGCTTCTGGTGCGCCATGAGAAGGAAGTTCGTCGCTTGACCGCCGTTCTCAAGTTCATGCGCTGGTT
TGAGATTTTCTTTGCCTTGCTTCCGCTGAAGCTTGTCTTCAAGCTCTTTTTGTTCTCTGAGGAATTCACCAAC
ACTATTGCTCTGCCGATGACGGCGTTGTTCCTCGGCACCGGTAATGCTACGCCTGAAGTCCCTGCGATTATGT
TTGAGAGGCTGTGTACTTCACCGACTTATGGAATGTGGTATCCGTCCGATAGAAACACCGTCATAAGCAACAA
ACCGCCCATGATTGTATTTCCAAAATTCTCAGAGTTCTACGAGACCTGGAGAAAGGATCTTGTTTCTCGCGGA
GTCACCGTACGGCTTTCAACAGAGTTGACCGAAATTGTCCAGAGGAATAAGAATGGCGTTGTTGTGAAGCTCA
AGGCGAGGACCCCGATGCCAGATCATCATAACCCCAACGGCGGTGATCCCGATGCACCCGTCGGCGAGGAGAA
ATACGACGAGATTGTGCTCTGCTGTCTCGCTGACACGGCAAAGAAAGTCCTCGGCAGGACCGCCACCTGGAAA
GAAAAGAAGGTCTTGGGTTCAGCAAAATTCAGCGACGATATTACAATCACTCACAATGATTCCGATTATATGA
AGAAACACTACGAGAACTTTTATCGTGATGATTTGGCTGTTGCGAACATCAACGGAACTGACCAATCGGATCG
TCTGGCGTTTGCAAAGACAGAATATCGACCCATGTACTACATCAAGATGTATCCTGAGGACAAGTCTAAGCTG
GAAATGTGCTTCGACTGCACGAACTACCAGAGCCAATTCCCTGAAAAGGTACCCTTTGAGCAACATGTCTTCC
AGACTATTTATCTCAACAAGGATCGGGACAGTAAGCTCTGGACTGATAATGAGATTGCCGAGGATAAGATCAT
TCGAAAGGACTGGTGGCATCAACTCTGTCACGGCTATACTCATTATCTGTTTGTCATCCCGTGGATGATGTTC
CTCAACGCCAAGAATCACACTCGCTTCGCAGCGTCTTGGACTCTTGTCAACGCCCACGAAGTCGCCGTCATGT
CCGGAATTGCAGCTGCCGTGGATCTAGGCGCCAACTACCCTGAAGACTTGGAGAACGATAAGTTTGCGTTCTT
GTGTTTCCGACTCTACTACCTTCTTACATATGGAAAATGGTACAGGAGACATTTTACGTCCAAGAAGTATTTA
AAGTCTCAGACCACCGAGAGTCAGGCTGCTGCTGATGGGAAGAGTTGGGCTACTGGGCTTTATGGCAGTGTCT
ACAAGGGACCTGGTGTTTCGGAGGTGGAGAGGAGTGCTTGGAGGGAGGAAATTAAGAAGGGATCTAGTACTGG
TAACTTGTCTTGA

FFUJ_10321

ATGTCTTCCATCGTAGACAGCGTCAAG**gtgccttcccaaattccctgttcactaccagtgattaacttgttag**
AACACCATCGCCGAGAACTTCGGTGGACCAGCCGAAAA**GCTCGCAACCCGTCAATTCTCGCTCGCCGAAACGC**
CCGACCTAATCAACAAAGTCGCCGTCGTCACCGGTGGCAGCGAAGGTATCGGCTACGGCGTCACACACACCCT
CCTCTCCCATAATATCAGCAAGCTCTTCATCCTCTCCATCTCCGAAGACGTTGTTAACGGCGCCAAAAAGTCC
GTCGCTGAGGAACTCGGCCAAGAGAAGGCTGATCGTACAGAGTGGATCCAGTGCGATTTGAGCGATTGGGCTG
AAGTGAAGAAAGCAGCTGAGCAGATCAAGAAGTCGACTGATCGCTTGGATATTCTTGTTAATAATGCGGGAAG
GGGAATCATGACGTATCAGCTTTCGAAGTATGGTGTTGATCAGCATATGGCTGTGAACCATTTTGGTCATGTT
CTTTTGACTTCTTATTTGCTTGAACTTTTGAAGAAGACGGCTGATGAGCATGGGACAGTGAGGATTGTTAACC
TGGCTTCTAATGCGCATCAGGGGGCGCCCAGCGATGTCAAGTTTGAGAGTCTGGATGAGCTGAACCAGGATCT
TGGACCGAATCCTCAGTATGGGCGATCCAAGCTGGCTGCTATTTTATACAGTCGGTATTTGGATCGTCATGTC
ACCAAGGCTGGAAATCCCAAGATCTTGGTGAATGCCACGCACCCCGGATTCGTCAGCACCAGACAGAGTGTTG
AGCATATTCATGAAGC**gtgagtgcacctactctatcactcttaacacactcactgacaaatactag**ATATCCG
CTTGCTGGATATGCCATGTCGATTGGCATGGAGCCCTTCAAGAAGGATCAATTCCAGGGCGCCACTAGTTCAG
TCTTCGCTGCGACCATCACTGATAAGTCGGGACAGTATATCTGTCCTCCTGCTGTACCTGAGTCGGGTAATGA
GCTTTCGCAGAATGAGCAGCTTGGCGAACAGTTGATGGAGTTGACGAGAGAGGTTATCAGCGACAAGTTTGAG
AGCTTTGACGATCGAAGGTTTTATTAG

FFUJ_01993

ATGTCTCTTCGCATCTCCCAATCTCTCCGCCGCGCAGCCCTGCGCCCC**AATCCATCAACCCTCCTCAGATCCC**
CAATCATCGCCCACCGCTTTCAAAGCACCCAGCAGAACAAAGACCTCCCCATCCTCTACTCCGCCCACGCCAA
AGTCGTCGGCGCCCGCAAGGGTCATGTCGAGGCAGAGTCTCTCAACGTCGACCTCACCATGTCCAAAGCCCTC
GGCGGCCCCGGCGACGCAGGCAAGACTAACCCGGAGGAGATGTTCGCCGCTGGCTACGGCGCGTGCTTCCAGT
CTGCCATGAATGCCGTGGCCGCCAAGGACGGCATCACTATGCCCACTGCGCCTGAGGACAGTATTGTTGAGAC
GACGGTCCATCTTGTTGGTGATATGAAGAAGCTTGATATGCATCTTCGTGTGGATATGAAGATTATGGTCAGG
GGATTGGAGAAGGAGAAGATAGAGTCTATTGTTGAGAAGACGAAGAAGATTTGTCCGTACAGTAGGGCCATTG
AAGGCAACGTCTGGACAGAGTGCACGGTTGTTAAGCTGGATTAA

FFUJ_03407

ATGTCTCTCAACTCCAACCAACTCGACCCTCGCCAGCATCCTCCCCGGGCCCTTGGCAATGCTATGGCGCTGA
TACTCACATCCCCGAAGCTAATCACGCAGGCCTCATGTTCTTCAAAGATCACGGAACTCCCCAAGGATGGCGC
TTCAACCACGGCTACGGATGCCAAACCAATTGG**gtaaactccaacgaagagcttgtccaagcacgatcagaag**
cagtttactggctgtgactccatataaatgtgccgcgaggctcctgattactcgaacagAGATCTTTGGGGGG
CCGTTGAGAAGGGTGAGGAGATTGAGAGGACAGCGCATGTACTGGTCATGGATCCTAAACAGGCGGATCCCGA
CAAGCTTGGGTTTGATCCTTTTGATGTCACAAAG**gtttggccgcaatcgcagttccgatgaaggattttggaa**
gattggttcttaacaagaacccgggaactaccatcacaatatggaacagGTTGCGTTTTCGCCTAGCAGTATG
GTTCCTGGTATTGAGGACTCTCCTGACCCTCTTCTTCAGTTCCACGTGTTCTTCTACCGCGACGCTCAGTACC
GTCGCATCGGTGTCAATCTG**CTCCCAACAGCTTCGCTTACAAGTTCAGAGCTCGTGTCGCTGAGGCGCCGTAT**
CAGGTCAGCGACAACATCGAAAGCAGGGTTAGTCACTGTTGACTTGAGG**gtaagaagaattgctacgatcaag**
cgaaggatctgtggacgaggattatgagtgagcagGAGAAGAAAAATACTCGCTACAATACAGCCAAGGGTCT
TCGCCGCATCAAGCTCCCGAGATCAAG**gtaagcaatcaagctattttgaataagacttcagctgacagaagta**
tagAGCAAGTGTTTGGCGCAGGTGTAAAACATCTCGGAGGATTATGCGCAAGGCATTTATGATCTTCTGGGTC
AACATCAGGTTGAGTTCCCCAAAGTCAAGGGACTGA

FFUJ_11802 (*carRA*)

ATGGGCTGGGAATATGCCCAAGT**gtacgtattctcggcgttgttgagggggcgtttaactcatcccttaatag**
GCACCTGAAATACACGATACCGTTTGGTGTTGTTTTGGCGGCGGTTTACAGACCGTTGATGTCACGGCTGGAT
GTTTTTAAGCTTGTGTTTTTGATAACGGTGAGTTTTTTCTGGGTGGTTAAAGGACTTGAAGCTAATTCGTGCA
GGTTGCTGTTGTTTCTACCATGTATGTTGATTTTGAGGGGGGTTTTGTTGAGGTTGACTAACGCAGACAGCCC
CTGGGACTCGTATCTCATCAAAAATCGAATATGGACTTATCCTCCTGGTGTCGTGGTTGGTCTGACAGCATGG
GATATCCCAGCTGAAGAATTATTTTTCTTCGTCATTCAGACTTTCAACACCTCTCTACTCTACATGATTCTCA
GCAAACCAACATTTCACCCTATTTACCTGTCAAAGAAGACGGGCTGGGGTAAAATTGCTGGCCAGATTCTTTT
CGCATCAGCTATTATTTTCGGCCTCGTTTCTGTTTCCTCTGGTGGCGAGGGGATGTATATGGGCCTTATCCTC
ATTTGGGCTTGTCCATTCCTCTTGTTTCTATG**gtaatactcaacttgagagactatttcacgctagatatgct**
aaccattgacagGTCAATTTCTTACCAGTTCATAGTGAACTTGCCATGGACGAATACTGCGCTTCCAATTGCT
CTGCCAACGCTGTATTTGTGGGTTGTTGACACCTTTGCGTTGCGACGAGGAACATGGAGTATCACTTCTGGAA
CCAAATACGGAGTCGTTTTGTGGGATGGCCTGGAAATTGA**gtaagcgaccattctcctgagagatgtgactat**
tctgaccatttccagGGAGGCTGTCTTTTTCCTTCTCACCAACACCTTGATTGTGTTTGGCCTCATCGCTTGT
GACAACAACCTAGCTATCCTCGATACATTTCCTGAGCATTTCCCTCGGACAAAAGGAGTGCCCAGCTTGCTCA
CAATCATCCGAACTCTGATTTTACCTAAAGAGAAGTATGATGAAGAGCGCATTCAAGGTCTTGTCAGCGCAGT
TGCTTTACTTCGCAAGAAGAGCCGAAGCTTCTATCTCGCAAGCGGCACTTTTGAGGGAAGACTCCGCATTGAT
CTGATTCGTTTGTATGCGTTCTGTCGAGCTGCCGATGATCTCGTTGATGAGGCTCCATCTGTTGATGATTCCA
GAGCCTCTATCGAGAAGCTGAGAAAGTTTCTTGATCTCGCTTATGAAGAGAATCAAGAGGAACCTTCTCAGAG
ACTACGAGAATATGTCACGTCCAGCATCCCGGAGATGTTCCACATGGCTCTTCTCCAACTGCCTACTTATTAT
CTGCCAAAACAACCTCTAGACGATCTCCTTAAAGGCTTTGACACGGATCTTCTCTTTGACAGAAAGTCCGGCG
CATTTCCTATCGAGACCACTGAAGATCTTGACATCTACGGAAGTCGTGTAGCTGGCACTGTCGCAGAACTTTG
CAACCACCTTATTCTCTACCATACCC**CAGAAGCTGTTCCCGAAGACATCCAGCGCGAAGTTGTCGCTTCAGGT**
CAAGAAATGGGCATCGCACTTCAATACGTCAACATCGCTCGCGACATCAAAACCGACGCAGAAATTGACCGCG
TGTATCTTCCCCTCTCCTGGCTCAAAGAAGCCCAACTCACTCCTGAAGATGTAATTCAACAACCCCATGGTCC
TACCATTGAAGCTCTCCGTCATAAATTGCTTGACCGTGCTTTTGAAAAGTACAACATGGCAAAGGGCGCTATT
GATAAGCTCCCTTCAGAAGGAAAAGGTCCAATCCGCGTTGCTGTTGAGAGCTACATGGAAATTGGACGCGTTC
TTAGAGAGAAGGGTCCTACGATGAAGAAGGGCAGGGCTACTGTTCCTAAGATGAGACGAATTCGTGTTGCATG
GTCTGCATTGAACAAATAG

FFUJ_11803 (*carB*)

ATGAGCGACATTAAGAAATCTGTTATTGTTATTG**gtatgttactcctaatatcatgagcatgagaaggtattg**
actgacagttaccagGTGCTGGTGTCGGTGGTGTTTCTACTGCTGCGAGACTTGCAAAAGCTGGCTTCAAAGT
CACTATCCTCGAGAAGAATGACTTTACCGGTGGACGTTGCTCTCTAATCCACAACGATGGCCAC**gtacgtctt**
ccttctcactctttttacgcccactaaccttgtcagCGCTTCGATCAAGGTCCATCTCTTCTTCTCCTCCCTC
GCTTCTTCCACGAGATCTTCCAAGACCTAGGAACATCTCTAACTGCTGAGGGCGTTGAGCTTTTGAAATGTGA
ACCCAATTACAACATCTGGTTCGGCGACGGTTCATCTTTTGAGATGTCTACTGATCTCACCAAGATGAAGAAA
GCTATCGAAGCCGTTGAAGGTATCGATGGTTTTGAGAGGTACCTCGGTTTTCTTCAGGAGTCGCATCGACATT
ATGAAGTCAGTGTTGAGTCTGTGCTGCGAAGAAACTTTCCTAGTATTTTGAGCTTGGCGAGACCTGAGGTGCT
GTTCAATCTGTTCAATATTCATCCCCTTGAGAGTATCTGGACGAGAGCGAGCAAGTACTTCTGGACTGAGAGG
TTAAGAAGAGTCTTTACATTCGGAAGCATGTACATGGGCATGAGTCCGTTTGATGCGCCAGGAACGTATAGCT
TGCTTCAGTATACTGAGCTTGCTGAGGGTATCCTATATCCTCGAGGTGGATTCCACAAG**gttagtcttgcaac**
ccgacatgataggtttctcactaacagcttcagGTCGTTGAGGCACTGGTTAACGTTGGTCAGCGTC**TCGGTG**
TCGAGTACCGTCTCTCCACTGGCGTCAAGTCCATTTCTATTGACCAAGCAACCGGCAAGGCAAACGGTGTCGT
TCTGAGCGACGGAACACATTTACCTTCAGACATTGTCATCTCAAACGCCGACCTTGTCTATACTTACAACAAC
CTCCTTCCTAAGACCAGCTACGCAGACTCGCTATCAAAACGAGAAACCTCCTGCAGTAGTATCTCTTTCTACT
GGTCTGCTTCTAAGATCGTTCCTGAACTCAACGCTCACAACATCTTCCTTGCGGATGAGTACCAGGAGTCTTT
TGACAGCATCTTCAAGGAGCACCTCATTCCTTCAGAACCATCCTTCTATGTCAATGTTCCTTCACGCATCGAC
CCTTCAGCTGCTCCTGAAGGTAAGGACTCTATAGTCGTCCTTGTACCCGTTGGCCATCTTCTGTCAGATTCTG
AAGGAACACATCGTGGTTTGTCCAAGTCTGGAAATTCTGGTGGCCTTGAAACAAGCCAGGACTGGGATAAGAT
GATCTCTCTGGCCCGTGACACAGTCATCGCAACAATGCGTGCGAGAATAGGCGTTGATCTTGCTCCTCTCATT
GAAAACGAAATCATCAACACTCCCTTCACGTGGCAAGAGAAGTTCAACCTCGACAAGGGTGCTATTCTTGGCT
TGAGCCACTCCATCATGAATGTTCTCGCCTTCCGACCTGGTACTCAGCACTCCAAGTACAAGAACTTATACTT
TGCTGGTGCCAGCACACATCCTGGTACTGGTGTTCCTGTCTGCATTGCTGGTAGTAAGATTGTTGCAGAGCAG
ATTCTCAAGGATTCAGGTTTCAAGAACAACCAGATCCCCTGGGCTCAGGATACTACCAAGTCTCCCAAGGGTG
GACTGGATAAGATGAGCGATTCGTCTTTGACTCTGTTCCAAGGGTTCTTGGGGGCTCTGGTTGCGATTTTGCT
GGCTTATTATTATCTTGTCATTGCTGCGAATTAG

FFUJ_13490

ATGGCTCCCATCAAGGTCGGCATCAACGGTTTCGGCCGTATCGGCCGTATCGTCTTCCGCAACGCCGTCGAGC
ACCCTGACATCGAGGTTGTTGCTGTCAACGATCCTTTCATTGAGCCTCACTACGCC**gtaagtagaccccttgc**
gaactcggtcgatacttcaacaaccctacaatccacttctcgggagcttgtcgctttgtcacgaccgtttaaa
cgcgcgtttcgattcaactggagcaccgcacaatatcgtccataataatcattgtgccggaatatgtaattgg
catcgcgagctgtcgccagcttcgtgtcatggcatagtactagagagcgatgcttcatgtcccgccaagctac
ccctcactgccccacgccccatacaatatcacattcagctttgagcctcaagctaacgtcaactcccctacgc
agGTCTACATGCTCAAGTATGACTCTTCCCACGGTATCTTCAAGGGTGAGGTCGGCAACGATGGTAATGACCT
TGTCGTCAATGGCAAGACTGTCAAGTTCTACTCTGAGCGCGACCCCGCCAACATCAAGTGGTCCGAGACCGGC
GCCGACTACGTCGTCGAGTCTACTGGTGTCTTCACCACCATTGACAAGGCCAAGGCCCATCTTGCTGGTGGTG
CCAAGAAGGTCATCATTTCTGCCCCCTCTGCCGACGCTCCCATGTACGTCGTCGGTGTCAACGAGAACAAGTA
CGACGGCTCTGCCGACATCATCTCCAACGCCTCTTGCACCACCAACTGCCTGGCTCCCCTCGCCAAGGTCATC
AACGACAAGTTTGGTATCGTGGAGGGTCTCATGACCACCGTCCACTCCTACACTGCTACCCAGAAGACCGTCG
ATGGTCCCTCCGCCAAGGACTGGCGAGGTGGCCGTGGCGCTGCCCAGAACATCATCCCCTCCAGCACTGGTGC
CGCCAAGGCTGTCGGCAAGGTCATTCCTGAGCTCAACGGTAAGCTCACTGGCATGTCAATGCGTGTCCCTACC
GCCAACGTTTCCGTTGTCGATCTTACTGTCCGCCTTGAGAAGGGTGCTTCTTACGACCAGATCAAGAAGGTCA
TCAAGGAGGCCTCTGAGG**GTGACCTCAAGGGCGTTCTGGCCTACACTGAGGACGATGTTGTCTCCTCCGATCT**
CAACGGTAACACAAACTCCTCCATCTTCGATGCTAAGGCCGGTATCTCTCTCAACGACAACTTCGTCAAGCTG
GTCTCCTGGTACGACAACGAGTGGGGTTACTCCCGCCGTGTCCTCGACCTCCTGGCCCACGTTGCCAAGGTCG
ATGCCTCCAAGTAA

FFUJ_4397

ATGCGTGAGATT**gtaagtacctctttttagagttcgtattgtgctgttgcacgcgttgagtttaccgtgccc**
ctgattctaccccgctgggtggtggcagctcaacgacattgcacgatagctagcagctttaacctacctactc
tcaagacgaagaagctaatcagatattttctctacgatagGTTCACCTCCAGACCGGTCAGTGC**gtaagtgct**
catcgcttcctcaacgtcgcatgcgggggatgctcacaacgtttatcagGGTAACCAAATTGGTGCTGCTTTC
TGGCAAACCATCTCTGGCGAGCACGGCCTCGACAGCAATGGTGTCTACAACGGTACCTCCGAGCTCCAGCTCG
AGCGCATGAGTGTCTACTTCAACGAG**gtatgccttaacagtcaatgccaataatccccaagctcacacaacta**
gGCCTCTGGCAACAAGTATGTTCCCCGAGCCGTCCTCGTCGATCTTGAGCCTGGTACCATGGACGCTGTCCGT
GCTGGTCCCTTCGGTCAGCTCTTCCGTCCCGACAACTTCGTTTTCGGTCAGT**CCGGTGCTGGAAACAACTGGG**
CCAAGGGTCACTACACTGAGGGTGCCGAACTTGTCGACCAGGTCCTCGACGTTGTCCGCCGTGAGGCCGAGGG
CTGCGATTGCCTTCAGGGTTTCCAGATCACCCACTCCCTCGGTGGTGGTACCGGTGCCGGTATGGGTACTCTA
CTTATTTCCAAGATCCGCGAGGAATTCCCCGACCGAATGATGGCCACCTTCTCCGTCGTTCCCTCTCCCAAGG
TTTCTGACACCGTCGTTGAGCCCTATAATGCAACCCTCTCTGTCCACCAGCTGGTCGAGAACTCTGACGAGAC
CTTCTGTATCGACAACGAGGCCCTCTACGATATCTGCATGCGCACCCTGAAGCTGTCCAACCCCTCCTACGGT
GACCTCAACTACCTTGTTTCTGCTGTTATGTCCGGTGTCACCACCTGTCTCCGTTTCCCCGGTCAGCTGAACT
CCGACCTCCGAAAGCTCGCCGTCAACATGGTGCCTTTCCCTCGTTTACACTTCTTCATGGTCGGCTTTGCACC
TCTGACCAGCCGTGGTGCTCACTCTTTCCGCGCTGTCAGCGTTCCTGAGTTGACCCAACAGATGTTCGACCCC
AAGAACATGATGGCTGCTTCGGACTTCCGCAACGGTCGCTACCTGACCTGCTCGGCCATTTT**gtgagtgatcc**
cgattttgcacatggtaacaatttactgactttatccagCCGTGGCCGTGTCGCTATGAAGGAGGTCGAGGAC
CAGATGCGCAACGTCCAGAGCAAGAACTCGTCTTACTTCGTTGAATGGATTCCCAACAATATCCAGACAGCCC
TTTGTGCCATCCCTCCCCGAGGACTTACGATGTCTTCGACCTTCATCGGAAACTCCACCTCTATCCAGGAGCT
CTTCAAGCGCGTTGGTGAGCAGTTCACTGCCATGTTCCGACGCAAGGCTTTCTTGCATTGGTATACTGGCGAG
GGTATGGACGAGATGGAGTTCACTGAGGCTGAGTCCAACATGAACGATCTTGTCTCCGAATACCAGCAGTACC
AGGATGCTGGTATTGATGAGGAGGAAGAGGAGTACGAGGAGGAGCTCCCTGAGGGCGAGGAGTAA
